# Push–Pull Intercropping Increases the Antiherbivore Benzoxazinoid Glycoside Content in Maize Leaf Tissue

**DOI:** 10.1021/acsagscitech.4c00386

**Published:** 2024-09-24

**Authors:** Jakob Lang, Sergio E. Ramos, Linus Reichert, Grace M. Amboka, Celina Apel, Frank Chidawanyika, Andargachew Detebo, Felipe Librán-Embid, David Meinhof, Laurent Bigler, Meredith C. Schuman

**Affiliations:** † Department of Geography, University of Zurich, 8057 Zurich, Switzerland; ‡ Department of Chemistry, University of Zurich, 8057 Zurich, Switzerland; § Department of Ecology, Swedish University of Agricultural Sciences, 756 51 Uppsala, Sweden; ∥ Institute of Animal Ecology and Systematics, Justus Liebig University of Gießen, 35392 Gießen, Germany; ⊥ International Centre of Insect Physiology and Ecology, 40305 Mbita, Kenya; # Department of Zoology and Entomology, University of the Free State, Bloemfontein 9301, South Africa; ∇ Institute for Sustainable Development, Addis Ababa 1165, Ethiopia; ○ Department of Animal Ecology and Tropical Biology, Julius-Maximilians University of Würzburg, 97074 Würzburg, Germany

**Keywords:** UHPLC-MS, intercropping, agroecology, maize (*Zea mays*), push−pull technology, metabolomics

## Abstract

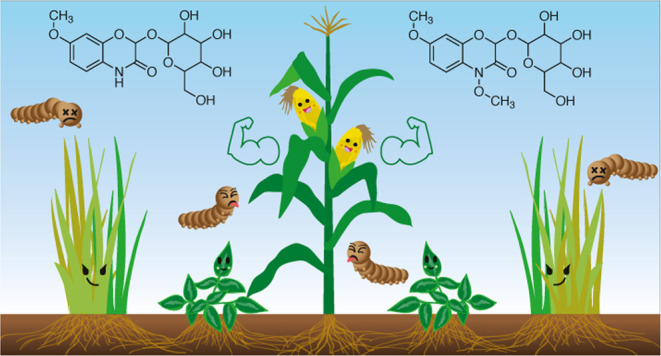

Push–pull technology refers to a promising mixed cropping practice for sustainable agricultural intensification, which uses properties of intercrop and border crop species to defend a focal crop against pests. Currently, the most widely practiced system uses *Desmodium* spp. as intercrop and Brachiaria or Napier grass as border crops to protect maize (*Zea mays*) against both insect pests and parasitic weeds. Several previous studies have demonstrated the efficacy of the push–pull system, but research on the underlying chemical mechanisms has mostly been limited to laboratory and glasshouse experiments that may not fully reproduce the complexity of the system under natural conditions. To address this limitation, we performed a large-scale study in farmer-operated push–pull maize fields in three east African countries. We compared maize leaf extracts from plants grown on push–pull fields with maize from fields employing conventional agricultural practices to assess the influence of push–pull cultivation on the maize metabolome. We identified two benzoxazinoid glycosides, which are known to have antiherbivore properties and were present in greater relative abundance in push–pull-cultivated maize leaves across three countries. Our data thus suggest that maize cultivated under push–pull has an increased resistance to herbivore attack compared to maize grown under conventional local agricultural practices.

## 1 Introduction

Push–pull technology refers to an agro-ecological technique that uses repellent properties of an intercrop (push) and attractive properties of a border crop (pull) surrounding the field for pest control.^1−3^ While there were early reports of push–pull systems in Australia and the United States,^4^ the best-established push–pull system has been developed for crop protection in sub-Saharan Africa.^5^ It involves intercropping the focal crop, usually maize or sorghum, with a legume of the *Desmodium* genus, which helps to reduce herbivore attack and suppresses the growth of the parasitic witchweed (*Striga* spp.).^2,6^ The technique was originally developed to address the biotic constraints faced by farmers in the region and has been shown to effectively improve crop yield without pesticide input.^3^

The proposed mechanism for insect pest control in the push–pull system is that the *Desmodium* intercrop emits volatile compounds that repel herbivorous insects (“push”), while a surrounding border grass emits attractive volatiles (“pull”).^7^ This guides the herbivores away from the focal crop and toward the border grass where they cannot complete their life cycle, thereby acting as a dead-end trap.^8^ Furthermore, suppression of witchweed in the system was linked to four flavonoids found in *Desmodium* root exudates that act in both pre and post parasite attachment phases to the maize.^9^ Other ecosystem services of the PPT include improved soil health by nitrogen fixation, improved soil organic carbon,^10−12^ and biodiversity conservation.^13^ While the reduction of herbivory rates and yield increases have been studied in multiple field trials,^12,14^ the molecular mechanism has mainly been investigated under conservative greenhouse or laboratory conditions, with little research conducted under field settings.^5^ The majority of publications on the mechanism behind the push–pull effect are focused on the influence of plant volatiles,^7,15,16^ but more recently studies on metabolites in greenhouse-grown maize planted with *Desmodium* or in push-pull conditioned soil showed notable changes in the abundance of various benzoxazinoids,^17−19^ which are a well-known class of compounds^20^ linked to plant defense.^21^ They are produced by various grasses, including agricultural staple crops such as wheat, rye, and maize^22^ and stored by plants in the glycosylated form. Upon tissue disruption the sugar is enzymatically cleaved,^21−23^ and some of the resulting aglycones such as 2-hydroxy-4,7-dimethoxy-1,4-benzoxazin-3-one (HDMBOA) have been shown to be active antifeedants and toxic to various herbivores^24^

Our aim was to contribute to the understanding of the chemical and biochemical mechanisms by which push–pull technology provides maize plants with better resistance against insect pests. By studying the metabolism of the focal crop directly in farmer fields, we aimed to identify molecular differences between crops grown under push–pull compared to those grown under conventional local agricultural approaches. We used an untargeted metabolomics approach that was adapted for sampling from ecological systems,^25^ which allowed us to study the aforementioned benzoxazinoids alongside other small molecules that may be linked to the effectiveness of push–pull in farmer fields.^26,27^ This allowed the analysis of a broad range of metabolites, which can be used to study biochemical interactions between plants and their natural environment, for example to evaluate the response to environmental stresses or diseases.^28,29^

We performed field sampling campaigns in three countries in sub-Saharan east Africa – Kenya, Rwanda, and Uganda–to collect leaf tissue extracts of 21 maize plants each from 37 push–pull fields which were paired with a set of 37 control fields following conventional agricultural approaches (such as maize monocrop or maize-bean mixed cropping^30^). Those samples were analyzed by ultrahigh performance liquid chromatography coupled to high-resolution tandem mass spectrometry (UHPLC-HR-MS/MS) and evaluated to determine metabolites which show higher abundances in maize from push–pull fields to identify potentially bioactive compounds.

## 2 Materials and Methods

### 2.1 Chemicals and Materials

Acetonitrile (MeCN) and isopropanol were obtained from *Biosolve* (ULC grade, Valkenswaard, Netherlands) and formic acid from *VWR Chemicals* (LC–MS grade, Dietikon, Switzerland). Ultrapure water (<2 ppb TOC) was produced using a Milli-Q Advantage A10 water purification system (*Merck*, Burlington, MA). For mass calibration, a 10 mM sodium formate solution and ESI-L low concentration tune mix bought from *Agilent* (Santa Clara, CA) were used. The 10 mM sodium formate solution contained 1 M NaOH (250 μL) and formic acid (50 μL) in 50% isopropanol (25 mL). Sample homogenization was done with micropestles from*Fischer Scientific*(Hampton, NH), extractions were performed using a 2:1 mixture of methanol with water (for molecular biology) from *AppliChem* (Darmstadt, Germany). All organic solvents used during sample extraction were obtained on-site and the supplier varies for each of the east African countries. For extractions performed in Kenya, methanol (EMSURE, analytical grade) was obtained from *Sigma-Aldrich* (Johannesburg, South Africa), chloroform for liquid–liquid extraction (reagent grade, 99%) from *Griffchem* (Nairobi, Kenya), and ethanol for cleaning of micropestles (100%, AR quality) from *Haymankimia* (Witham, United Kingdom).

### 2.2 Study Sites and Field Layout

**Figure 1:**
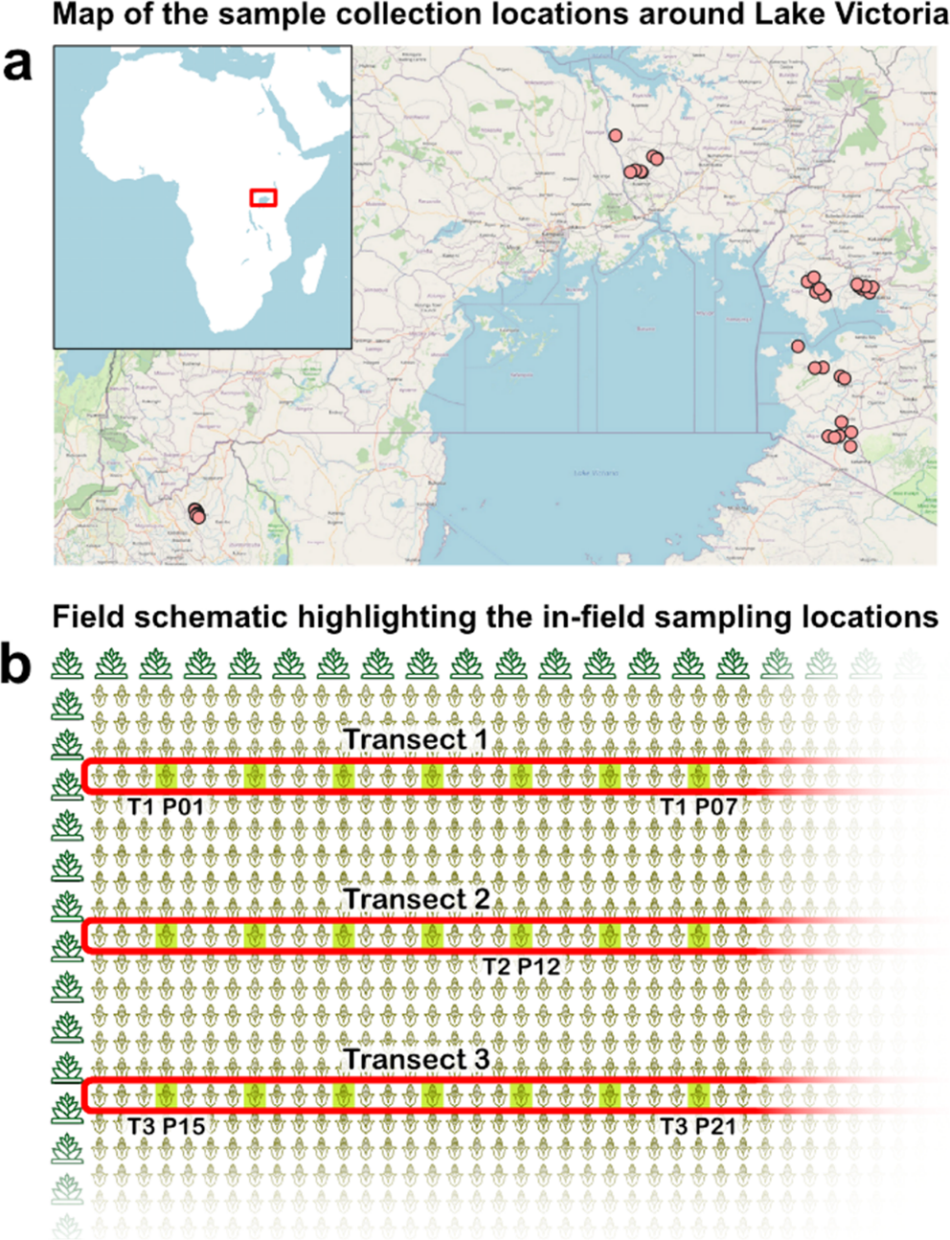
Map showing all field locations from which samples were collected in Kenya, Rwanda, and Uganda (a) and a schematic showing the sampling locations inside the fields (b). Samples were collected along three transects, where every fourth plant is used for sample collection. The sample naming is shown for a subset of plants to show the naming conventions. The map from *openstreetmap.org* is used in accordance with their licensing agreement.

Farmer push–pull fields were paired with comparable fields cropped under local conventional approaches. Fields were selected to match as closely as possible in terms of geographical distance, soil properties, plant age, field management (such as fertilization and pesticide use) and field size. Plant tissue collection was done during the short rainy season in 2022 (October–December) from 40 fields in eastern Kenya, 14 fields in Uganda, and 20 fields in Rwanda, always from an equal number of push–pull and nonpush–pull fields. The collection sites are listed in the Supporting Information Table S1 and shown in Figure 1a.

From each field, samples were collected following the layout shown in Figure 1b, where seven plants were chosen along three parallel transects each for a total of 21 tissue samples. The distance between transects was approximately 3 m, the distance between plants around 1 m (every fourth plant), and for smaller fields the distance was reduced to approximately 70 cm (every third plant) to maintain the same number of samples.

### 2.3 Sample Collection, Extraction, and Storage

Maize leaf tissue samples were taken using a previously published on-site extraction procedure.^25^ An extraction solution consisting of MeOH/H_2_O in a 2:1 ratio and camphorsulfonic acid (CSA) as an internal standard (20 ng/mL) was prepared, of which 200 μL were added to a 1.5 mL Eppendorf tube for each sample. Twelve leaf disks were collected directly into the extraction solution with a 6 mm diameter hole punch (Milian, Vernier, Switzerland), the tubes were thoroughly shaken and transported in a common household cooling box containing ice packs.

The leaf tissue was ground inside the Eppendorf tubes using plastic micropestles attached to a household electric drill. The micropestle tips had been roughened up by brief blasting with corundum (0.15–0.21 mm) out of a carbide nozzle (4 mm) at 8 bar, followed by thorough cleaning with deionized water, acetone, and ethanol before usage. After the leaf tissue was ground to a paste in the extraction solution, another 500 μL of the same solution was added before shaking thoroughly. Liquid–liquid extraction was then performed through the addition of 500 μL of chloroform to separate pigments and lipids, followed by thorough shaking. After letting the tubes rest for approximately 10 min at room temperature (28 ± 4 °C), the phase separation was completed, and the upper MeOH/H_2_O phase was transferred to fresh Eppendorf tubes. The extraction procedure was concluded within 30 h of collection and the samples were stored in household fridges (between 4 and 8 °C) and freezers (between −22 and −12 °C) until transport to Zurich, Switzerland. Samples were stored for a maximum of 3 months prior to analysis. The same approach was used at a subset of Kenyan field sites to extract leaf tissue of the *Desmodium* intercrop. Furthermore, tissue extract samples of the various border grasses used in the different push–pull generations were collected from demonstration fields of the International Centre of Insect Physiology and Ecology (icipe) using the same method.

### 2.4 UHPLC-HR-MS/MS Measurements

Liquid chromatography was performed on a Vanquish Horizon UHPLC System by *Thermo Fisher* (Waltham, MA) built from a Vanquish binary pump H, a Vanquish split sampler HT and a temperature controllable Vanquish column compartment. Chromatographic separation was achieved on an ACQUITY Premier CSH C18 Column (130 Å, 1.7 μm, 2.1 × 50 mm, *Waters*, Milford, MA) at 30 °C. Eluent A consisted of H_2_O + 0.1% HCOOH and B of MeCN + 0.1% HCOOH. The solvent flow was kept at 0.6 mL/min with the following gradient: (i) 5% B isocratic from 0.0 to 0.4 min; (ii) linear increase to 35% B until 2.8 min; (iii) linear increase to 75% until 3.2 min; (iv) linear increase to 100% B until 3.3 min, (v) holding 100% B until 4.4 min (vi) back to the starting conditions of 5% B until 4.5 min; (vii) equilibration at 5% B for 1.1 min until the next run.

A timsTOF Pro hybrid quadrupole-time-of-flight (QTOF) mass spectrometer equipped with trapped ion mobility spectrometry (TIMS) produced by *Bruker* (Bremen, Germany) was connected to the Vanquish UHPLC system and was used to acquire MS/MS data in positive and negative ESI ionization mode. The data was recorded without ion mobility and the scan range was set to 20 to 1350 *m*/*z* at a 12 Hz base acquisition rate. Mass calibration was performed using the *Agilent* low concentration tune mix (13 compounds in acetonitrile, part number G1969-85020) prior to analysis. For additional mass accuracy, a calibration segment was programmed from 0.05 to 0.15 min at every UHPLC run with the help of a 6-port-valve with a 20 μL loop which contained a solution of 10 mM sodium formate. Fragment spectra were acquired using the data-dependent acquisition mode (AutoMS/MS) employing 20 and 50 eV fragmentation energies.

### 2.5 Software and Data Treatment

Instrument control was done using Hystar (*Bruker*, version 6.0) and otofControl (*Bruker*, version 6.2) followed by data treatment (detailed below) in MetaboScape (*Bruker*, version 2022b). Figure plotting was done using Python (version 3.8.5) in the Spyder IDE (version 5.0.3) using the libraries pandas (version 2.0.3), seaborn (version 0.12.2), and bokeh (version 2.3.2). The molecular classification was done using Canopus^31−33^ in combination with Sirius^34,35^ (version 5.8.0) and CSI:FingerID^36−38^ was used for compound annotation of peaks that were not annotated by MetaboScape.

Peak extraction was done using MetaboScape’s 3D workflow. To be included in the final table, a feature was required to be present in at least 10 samples across the full data set and additionally, in 75% of samples in at least one field site. For the peak picking an intensity threshold of 1500 was used with 7 points across the peak. For recursive peak picking, the number of points per peak was reduced to 5. The internal mass calibration function was set to use sodium formate cluster signals from 0.05 to 0.35 min and no batch correction was applied. The peak tables of positive and negative polarity were then merged (3 ppm mass and 7 s retention time tolerances), the resulting table normalized by the signal of the internal standard CSA (2.7 min, [M-H]^−^, 231.0694 *m*/*z*) to compensate for transport and sample handling variations.

### 2.6 Isolation and Identification of HDMBOA-Glc

2-O-glycosyl-4,7-dimethoxy-1,4-benzoxazin-3-one (HDMBOA-Glc, see Figure 3) was isolated from a pooled mixture, where the samples that showed the highest abundance were pooled together. The purification required three steps, starting with a semipreparative RP-HPLC setup using UV-assisted automated fraction collection. This first purification step resulted in a mixture of approximately 10 different compounds, which was then further purified using the same analytical UHPLC setup used for the UHPLC-MS analyses. The second purification step was done using an ACQUITY Premier HSS T3 column, but as the column was not able to fully separate HDMBOA-Glc from a contaminant, a third purification on an Accucore Phenyl-X column was required to obtain the pure compound. A total of 400 μg of HDMBOA-Glc were obtained after the three-step purification. The detailed HPLC methods are described in the Supporting Information and Figures S18–S23 show the solvent gradients and chromatograms of the purification process.

The purified compound was dissolved in MeOH-d4 (concentration 0.4 mM) and used for NMR analysis on a Bruker AV-600 MHz instrument equipped with a TCI CryoProbe. The following experiments were included: ^1^H, heteronuclear single quantum correlation spectroscopy (HSQC), correlation spectroscopy (COSY), heteronuclear multiple bond correlation (HMBC), and total correlation spectroscopy (TOCSY).

## 3 Results

### 3.1 Metabolome Differences between Push–Pull and Conventional Maize

A direct comparison of all collected samples by principal component analysis (PCA) as shown in Figure S1 reveals that the samples are grouped by country. This is not surprising given the variation in sampling and extraction by different researchers using different material suppliers, as well as environmental variation and possible variation in landraces and other aspects of cultivation practice across countries. However, the countries mainly separate along PC2, which explains 11.6% of the variance, while PC1, describing 24.5% of the variance, contains most of the variation within countries. Using a single country data set, PCAs do not show clustering of samples from push–pull versus conventional fields (Figures S2–S4), indicating that other sources of variation may have a greater effect on the overall leaf metabolome.

**Figure 2:**
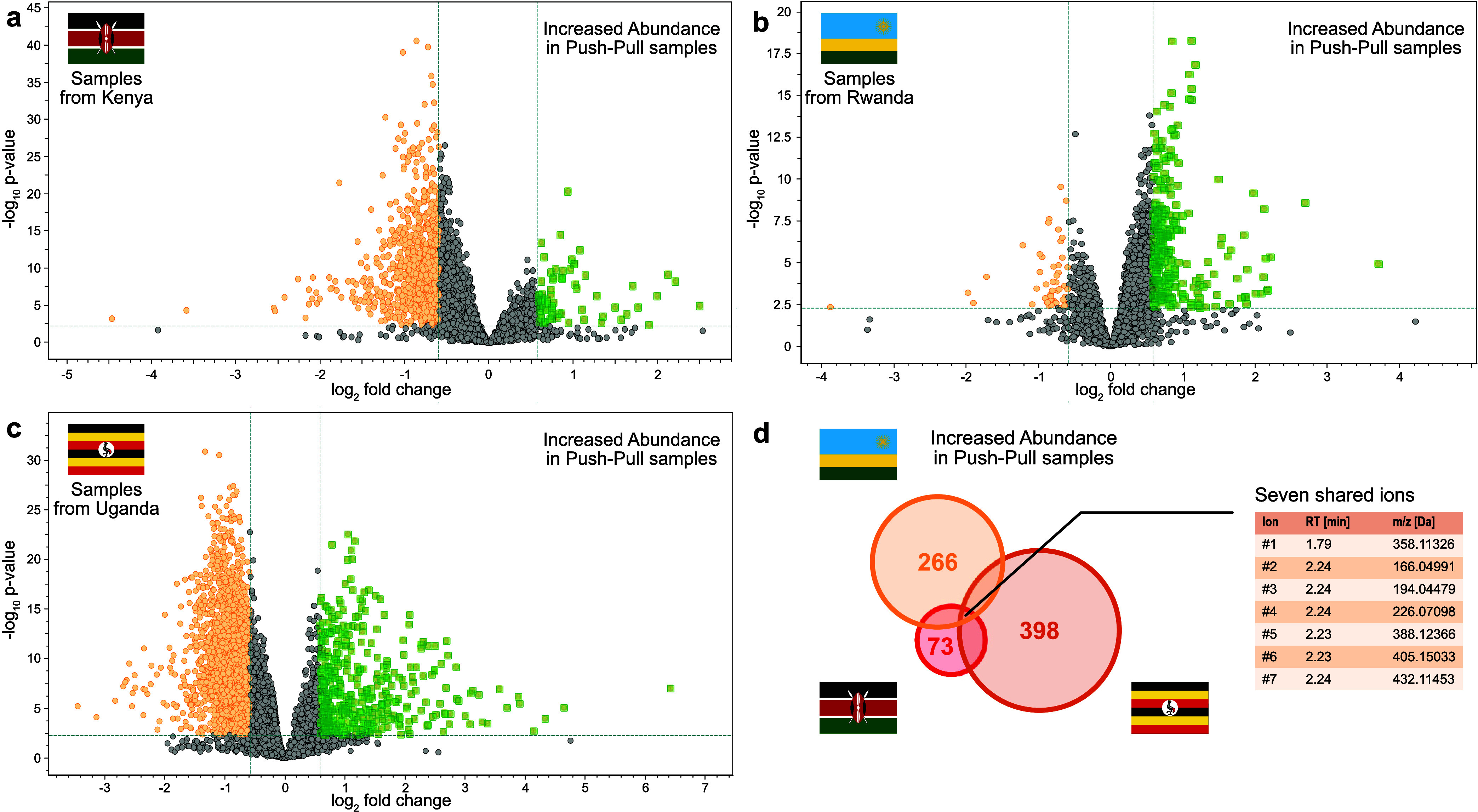
Volcano plot analysis of the metabolic differences between conventional and push–pull maize by country ((a) Kenya, (b) Rwanda, (c) Uganda). The features shown in green show an increased abundance in push–pull fields by a factor of at least 1.5, with a p-value <0.005. Those features were then used to determine the international consensus, shown in the Venn diagram (d) which resulted in seven features to be identified.

We thus employed a *t* test as to identify features differing between push–pull and conventional fields, which is visualized in volcano plot form in Figure 2a–c. Due to the large variance in the data set and large number of features, a significance cutoff of 0.005 was chosen instead of the commonly used 0.05, which was combined with a minimum 1.5 fold increase in abundance in samples from push–pull fields compared to the conventional fields to determine potential compounds of interest. The three countries were evaluated separately, resulting in 398, 266, and 73 features identified for the Ugandan, Rwandan, and Kenyan data, respectively. Of those, seven features are shared among all three evaluated countries (Figure 2d), which were then evaluated further to identify the metabolites of higher abundance in push–pull fields.

### 3.2 Identification of Compounds of Interest

**Figure 3:**
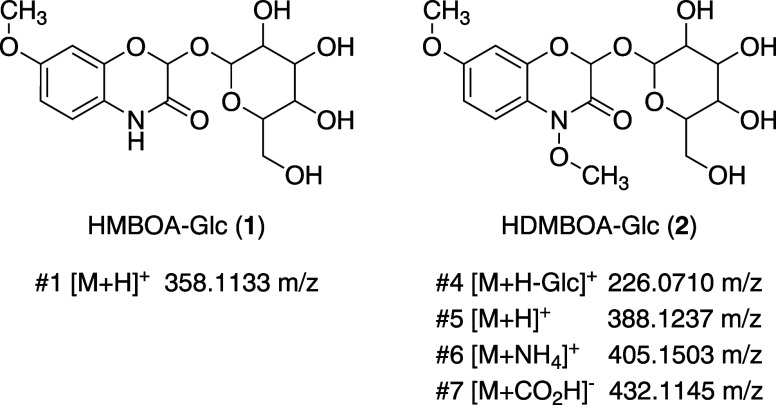
Structure matches for five of the seven target features following the CSI:FingerID substructure matching with public libraries. HMBOA-Glc (**1**) was assigned through library spectra matching, while multiple adducts and fragments of HDMBOA-Glc (**2**) were not matched to library spectra but shared the same chromatographic retention time and fragment classification.

One of the features could be annotated as 2-O-glycosyl-7-methoxy-1,4-benzoxazin-3-one (Figure 3, HMBOA-Glc, **1**) by matching to a library MS/MS spectrum^39^ while the remaining six features, without spectral library matches, shared the same retention time–an indication that they may originate from a single molecule. All seven features were then classified using a workflow based on Sirius, and Canopus, which assigned compound classes based on the ClassyFire compound class taxonomy.^31^ Additionally, the CSI:FingerID module of the Sirius workflow generates substructures based on a fragmentation tree, which is generated from the MS/MS spectra. These substructures are then used to propose molecular structures found in databases such as KEGG or PubChem that show a combination of those substructures.^38^

The classification supports the result of the spectral library matching of **1**, with the assignment of the classes of hexose glycoside and benzoxazinone. Furthermore, the fragmentation tree-based structure proposal matches the spectral library annotation, with the second highest rated structure proposal having a shift of a methoxy group from position 7 on the benzoxazine core to position 4.

The annotations of the unknown features, which share the same retention time, indicate that they all originate from 2-O-glycosyl-4,7-dimethoxy-1,4-benzoxazin-3-one (Figure 3, HDMBOA-Glc, **2**) as features #5, 6, and 7 were assigned as various adduct combinations of **2**, while feature #4 was annotated as the deglycosylated 2-hydroxy-4,7-dimethoxy-1,4-benzoxazin-3-one (HDMBOA). The annotations of features #2 and 3 match substructures of HDMBOA, and the full annotation result are shown in the Supporting Information (Table S2).

**Figure 4:**
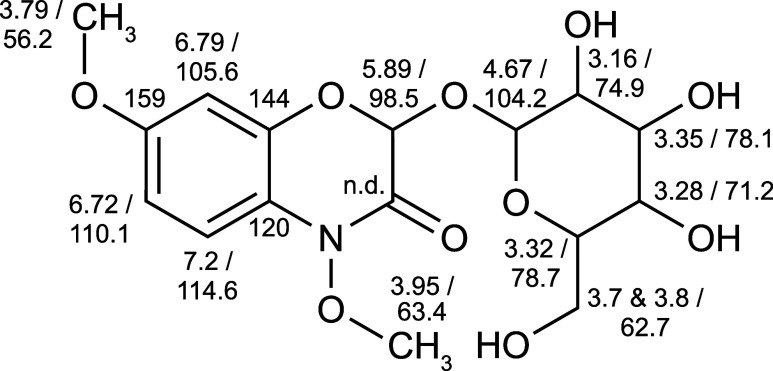
Structure of HDMBOA-Glc (**2**) with the measured ^1^H and ^13^C NMR shifts in ppm. Carbon shifts were determined by HSQC, HMBC, and TOCSY experiments as the low concentration of the sample did not allow a 1D-^13^C measurement. n.d. = not detected.

To improve our annotation confidence, the peak at the retention time of 2.24 min (containing target features #2 to #7) was isolated in a three-step HPLC purification, which resulted in 0.4 mg of pure **2** which was then used to verify the structure by NMR (see: Figures S24–S29). Due to the low concentration of the NMR sample, one-dimensional ^13^C spectra could not be recorded and we did not detect a signal to assign to the carboxyl moiety in the HMBC or TOCSY measurements. All other expected signals were detected and assignable and the shifts are shown in Figure 4.

### 3.3 Presence of Target Features

**Figure 5:**
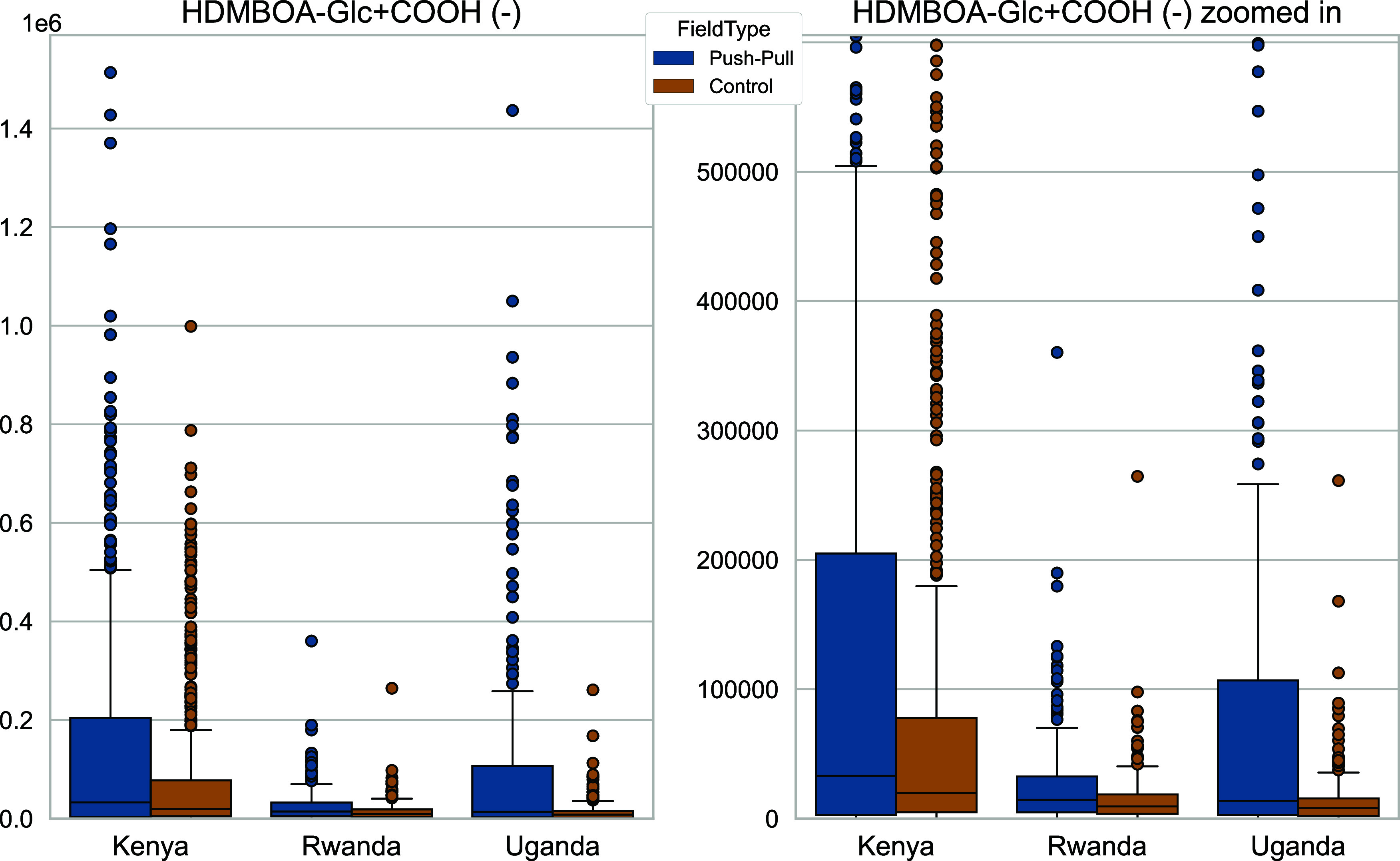
Boxplot of the abundance of target feature #7 (HDMBOA-Glc, [M + COO]^−^ ion, 432.11453 *m*/*z*) by country and field type. The right side shows a zoomed view to better visualize the differences (for each country p-value <0.005, see: Supporting Information Table S3) in the samples from Rwanda and Uganda. Feature #7 was selected for this visualization, because it showed the largest mean signal intensity, but all other target features show similar patterns. Sample counts: Kenya 661 push–pull and 661 control samples; Rwanda 286 push–pull and 286 control samples; Uganda: 209 push–pull and 210 control samples.

The features with shared greater abundance in push–pull maize nevertheless show pronounced differences in relative abundance among countries, with samples from Kenya showing the highest abundance, followed by samples from Uganda, while samples from Rwanda have the lowest signals. Overall, there is a large range of relative abundance, which is indicated by the large number of samples visualized as outliers in Figure 5. This variability is often caused by field-to-field variation, where samples collected from individual field sites can have a median abundance 10-fold higher than the national median (best seen in Figure S17). The patterns shown in Figure 5 are also observed for the other target features, both in terms of national differences and the outlier characteristics caused by selected field sites as shown in Figures S5–S17.

## 4 Discussion

### 4.1 Large-Scale Field Metabolomics Study of Intercropping’s Molecular Effects

Intercropping systems such as push–pull technology are frequently evaluated in terms of yield changes,^12,40^ herbivore damage,^41,42^ or economic viability.^43−45^ However, the underlying chemical and biochemical mechanisms are challenging to analyze, and thus are rarely studied.^5^ The few studies that have explored such mechanisms in push–pull have done so under laboratory or greenhouse conditions,^9,15−17,19,46^ thus reducing the number of environmental variables that influence the analyses. Here, we showed the results of the first large-scale plant metabolomics study of farmer-run push–pull fields in which we cover a total of 74 fields across three countries.

The sample collection and extraction were performed by different researchers following the same protocol with slightly different materials. The solvents for extraction were acquired locally and thus were of different brands and purities. This, alongside the tissue homogenization process being performed by different researchers, may contribute to the national clustering seen in Figure S1. Furthermore, the data shows large field-to-field variation, which is best seen in Figure S17, which likely is linked to variables such as soil composition, rainfall, plant age, or cultivar. Farmers often use local maize varieties,^47^ making identification of the precise cultivar challenging, however, these are commonly grown by farmers because they are seen to be more reliable compared to the purchasable standardized varieties.^48^ Furthermore, there are regional differences for the onset of the rainy season,^49^ which influences the time of seeding and thus plant age. The field-to-field variation thus is a direct consequence of sample collection from farmer fields, which could not be resolved without interfering with the livelihood of local farmers. Finally, these farmers’ fields are distributed across multiple environmental gradients, which likely contributes to variation both within and among countries.

Due to the large variation in our data set, we approached the selection of our target features conservatively. We focused on features that showed an international consensus of at least 50% increased abundance with a more restrictive 0.005 *p*-value maximum. While the single-country data sets showed up to 398 features matching those selection criteria (out of around 9000), the three-country consensus comprises only seven features, which are attributed to two molecules. This allowed us to focus on the metabolites with the highest probability of being relevant in the push–pull system and perform structure elucidation. However, this approach also means that there may be multiple other interesting metabolites in our single-country target lists that are not discussed in detail here.

### 4.2 Bioactivity and Origin of the Target Molecules

The target molecules were identified by different approaches due to the limited amount of material available. The isolation of HDMBOA-Glc (**2**) resulted in barely enough material for an NMR analysis, while the peak identified as HMBOA-Glc (**1**) was present in notably lower quantities than **2**, which meant that an isolation for NMR analyses was not possible. As such we relied on the annotation by spectral library match and the confirmation from the fragmentation tree analysis with Sirius. Both identified molecules are from the class of benzoxazinoid glycosides, which are stable forms of benzoxazinoids that accumulate in plant tissue. Of our identified targets, HDMBOA (the aglycone of **2**) has been reported to act as a deterrent against herbivores, while also decreasing insect weight after feeding.^24^ The glycosylated form **2** does not show those deterrent effects, but the production of **2** can be induced both by herbivory and fungal infection.^24,50,51^

The increased abundance of **1** and **2** in samples collected from push–pull fields suggest a mechanism by which maize plants may show an increased resistance to herbivory. While insects are not directly deterred from feeding, damaging the maize will result in the deglycosylation of **2** to the toxic HDMBOA, which may stop the feeding of herbivores before they cause substantial damage to the plant. To verify that the increased abundance of the two benzoxazinoid glycosides is due to an increased production by maize plants, we screened the extracts of three border grass species and *Desmodium* intercrops from ten farmer fields (see: data availability). The two benzoxazinoids were not detected in those samples, consistent with the hypothesis that their greater abundance in push–pull maize is due to their greater production by the maize plants. The higher presence of **1** and **2** in samples collected from push–pull fields thus suggests that the maize plants are capable of producing some antiherbivory defense compounds in higher quantities, perhaps leading to increased resistance.

There are various possible mechanisms behind the production of **2** in push–pull. Although the induction of **2** by herbivore damage described by Glauser and colleagues^24^ would imply that plants in push–pull fields are more likely to be damaged than plants in conventional fields, this is unlikely, as multiple studies instead show a reduction in herbivore damage in push–pull fields cultivated by farmers.^6,52,53^ The biosynthesis of benzoxazinoid glycosides is also known to be under developmental control and a basal amount of these compounds accumulates in the absence of herbivore damage.^54^ A different possibility is that increased availability of nutrients allows the maize plants to produce more of **2**, which could be due to the ability of *Desmodium* intercrops to fix nitrogen.^11^ However, a clear assessment of the mechanisms that lead to the higher abundance of the two benzoxazinoids would require further research and there may be multiple other reasons for the increased production besides the two possibilities mentioned above.

The observed increase in abundance of two benzoxazinoid glycosides in push–pull fields seems to contradict a recently published greenhouse study, where planting maize in a shared pot with *Desmodium* led to a lower abundance of two benzoxazinoid glycosides in root tissue and of the benzoxazinoids MBOA and DIMBOA in leaf and root tissue.^17^ However, the differences in growth conditions and used plant tissues and the incomplete identification of the benzoxazinoid glycosides make a direct comparison between this study and our results challenging. Other studies of the influence of push–pull intercropping on maize metabolism found increases in the benzoxazinoids MBOA and DIMBOA and their correlation with insect resistance. However, they discuss the deglycosylated form,^18,19^ which might not be directly comparable to our detection of compounds **1** and **2**. Another key difference lies in the experimental conditions, as the field-grown plants we sampled were exposed to the environment, including herbivores, during the entire growth period, while plants used for the three studies discussed above were grown in greenhouses and were likely exposed to less or no herbivore pressure, thus not inducing the production of **2** as much in their samples.^24,51^

### 4.3 Limitations and Outlook

Our results indicate increased abundance of two benzoxazinoid glycosides in maize tissue grown under push–pull agriculture in farmers’ fields. It is important to mention that it is only possible to measure what was extracted. The sample preparation method we applied^25^ focuses on high-polarity, low-mass compounds, which excludes proteins, lipids, and many peptides that might have an influence in the push–pull system.

A challenge of the data analysis was the comparison of the different field sites and the field-to-field variation, which is a direct consequence of collecting samples from actual fields of local smallholder farmers. We approached the variation in our dataset by only focusing on the most promising subset of the overall metabolite profile, namely the use of a lower *p*-value of 0.005 combined with the overlap of significant features across the three evaluated countries. This left us with only seven features to be studied further, while single country data sets contained up to 398 features with an increased abundance in push–pull fields. It is possible that the feature lists of any individual country may include other bioactive metabolites, which may be relevant for the push–pull system in certain regions. Our analysis focused on the identification of the most promising candidates for the push–pull effect as a whole, while the lower priority features were annotated by spectral library matching and are listed in the publicly available data tables.^55^

While we were able to determine potential candidates that may influence the resistance of maize against herbivory, we only describe a pattern in this large observational study. We can only hypothesize why these features are found in higher abundances in plants grown under push–pull and their potential relationship to yield increases reported by other studies. However, with this work we highlight that the cropping system influences the measured abundances of two benzoxazinoid glycosides, adding to previous studies that found differences in the relative abundance of some benzoxazinoids in leaves of maize exposed to push–pull-conditioned soil or coplanted with one of the intercrop species.^17−19^ We hope that future studies will be able to present ecological and biochemical mechanisms underlying the upregulation of these compounds in push–pull maize and its influence on herbivory and crop yields.

## Data Availability

Processed peak tables of the maize extracts and raw data of the extracts of border grasses and *Desmodium* plants are available on Zenodo: https://zenodo.org/doi/10.5281/zenodo.11070442.

## Supporting Information Available

Figures 1–4: Principal component analyses of international and national data sets; Table 1: Field sites and dates of sample collection; Table 2: Target feature annotations by Sirius/CSI:FingerID; Table 3: Results of *t* test of target features by country; Figures 5–17: Abundance of target features by country and field type; Methods: Isolation of HDMBOA-Glc (**2**); Figures 18–23: Gradients and chromatograms of the isolation of **2**; Figures 24–29: NMR spectra of **2** (PDF)
