# Supplementary material for: Push–Pull Intercropping Increases the Antiherbivore Benzoxazinoid Glycoside Content in Maize Leaf Tissue
Source: ACS Agric Sci Technol. 2024 Sep 24;4(10):1074–82. doi: 10.1021/acsagscitech.4c00386 (PMC11497208; doi:10.1021/acsagscitech.4c00386)
Supplement: Supplementary file 1 — as4c00386_si_001.pdf [file as4c00386_si_001.pdf]

Supporting information to the manuscript:

Push-pull intercropping increases anti-herbivore benzoxazinoid glycoside content in maize leaf tissue

Jakob Lang*[1,2], Sergio E. Ramos[1,2], Linus Reichert[1,2], Grace M. Amboka[3], Celina Apel[4], Frank Chidawanyika[5,6], Andargachew Detebo[7], Felipe Librán-Embid[4], David Meinhof[8], Laurent Bigler[2], Meredith C. Schuman*[1,2]

Contact: jakob.lang@uzh.ch or meredithchristine.schuman@uzh.ch

Affiliations:

[1] Department of Geography, University of Zurich, 8057 Zurich, Switzerland
[2] Department of Chemistry, University of Zurich, 8057 Zurich,Switzerland
[3] Department of Ecology, Swedish University of Agricultural Sciences, 756 51 Uppsala, Sweden
[4] Institute of Animal Ecology and Systematics, Justus Liebig University of Gießen, 35392 Gießen, Germany
[5] International Centre of Insect Physiology and Ecology, 40305 Mbita, Kenya
[6] Department of Zoology and Entomology, University of the Free State, Bloemfontein 9301, South Africa
[7] Institute for Sustainable Development, Addis Ababa, Ethiopia
[8] Department of Animal Ecology and Tropical Biology, Julius-Maximilians University of Würzburg, 97074 Würzburg, Germany

Supplementary Information:

- Supplementary Figures 1-4: PCA plots of the merged international dataset and the three countries separately
- Supplementary Table 2: Target ion annotations by Sirius, Canopus, and CSI:FingerID
- Supplementary Table 3: Results of the t-test for the seven target features
- Supplementary Figures 5-17: Abundance of target ions by country and field type
- Supplementary methods: Isolation of HDMBOA-Glc – Purification Method description
- Supplementary Figures 18-23: Isolation of HDMBOA-Glc – gradients and chromatograms
- Supplementary Figures 24-29: NMR spectra of HDMBOA-Glc

1. Principal component analyses of the datasets

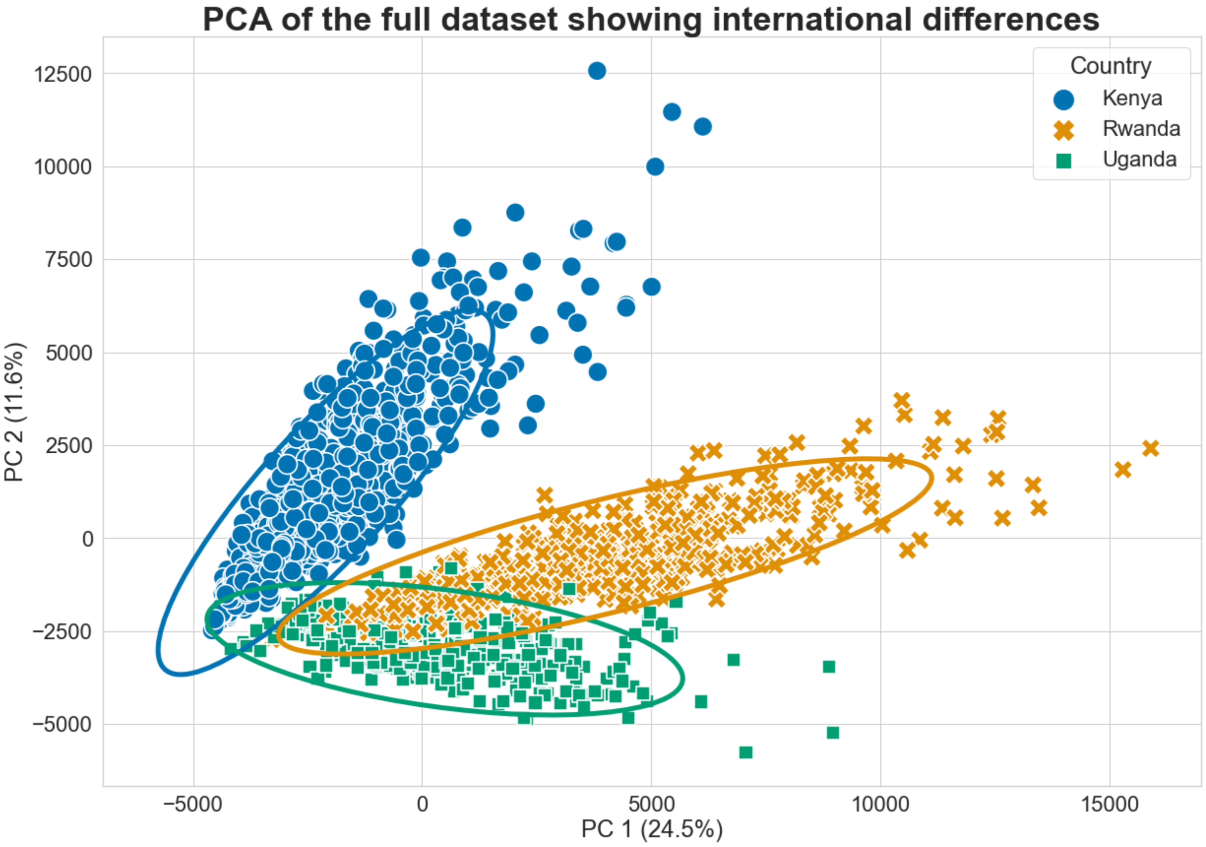

Fig. S1: *Principal component analysis of the metabolite data of samples from all countries. As the sampling was performed by different teams with different materials, those national differences are to be expected.*

Principal Component Analysis of Samples collected in Kenya - PC1 to PC5 shown

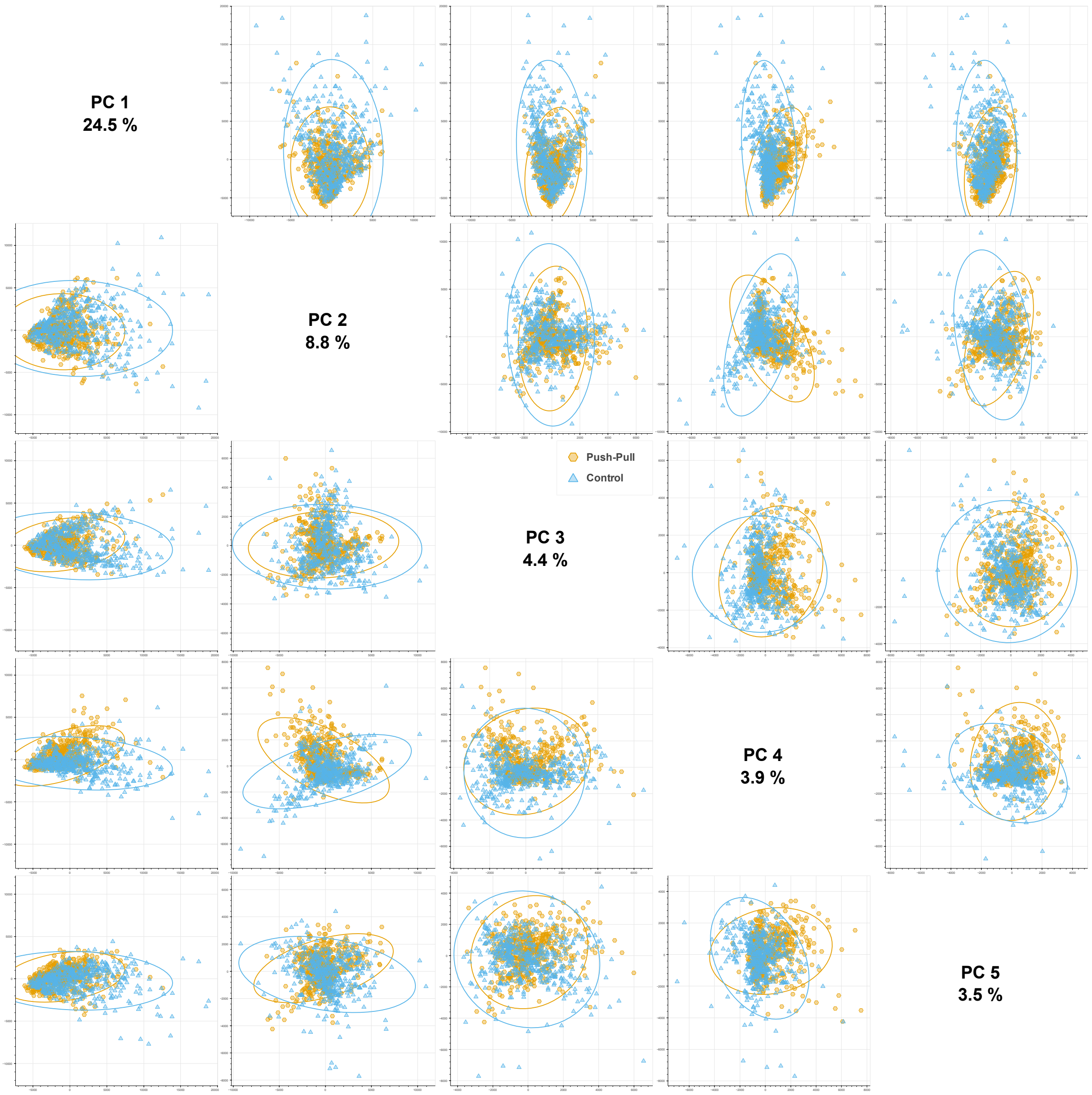

Fig. S2: *Principal component analysis of samples collected in Kenya. In the first five PC dimensions, no clear separation of samples from push-pull and control fields can be seen.*

Principal Component Analysis of Samples collected in Rwanda - PC1 to PC5 shown

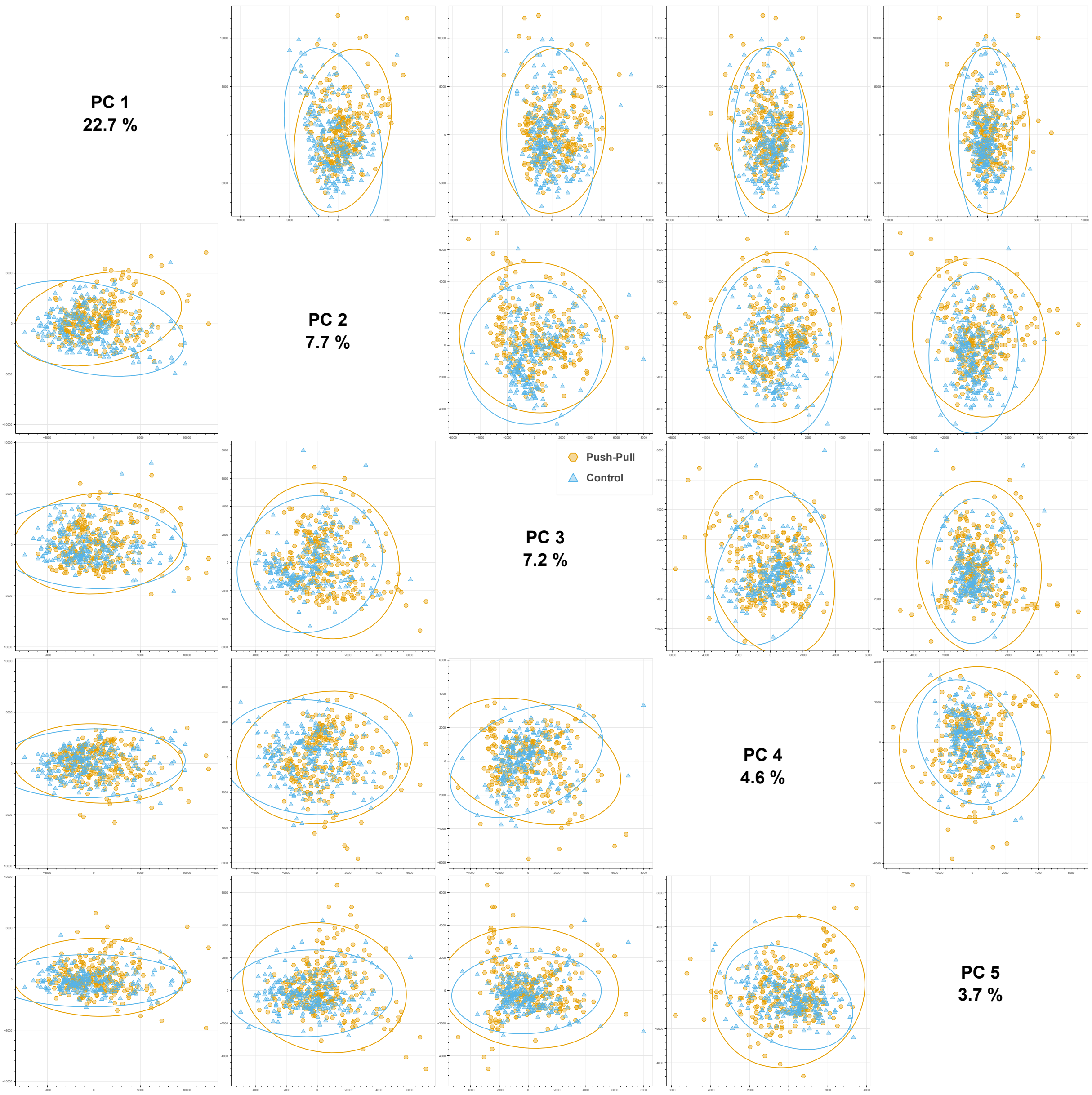

Fig. S3: *Principal component analysis of samples collected in Rwanda. In the first five PC dimensions, no clear separation of samples from push-pull and control fields can be seen.*

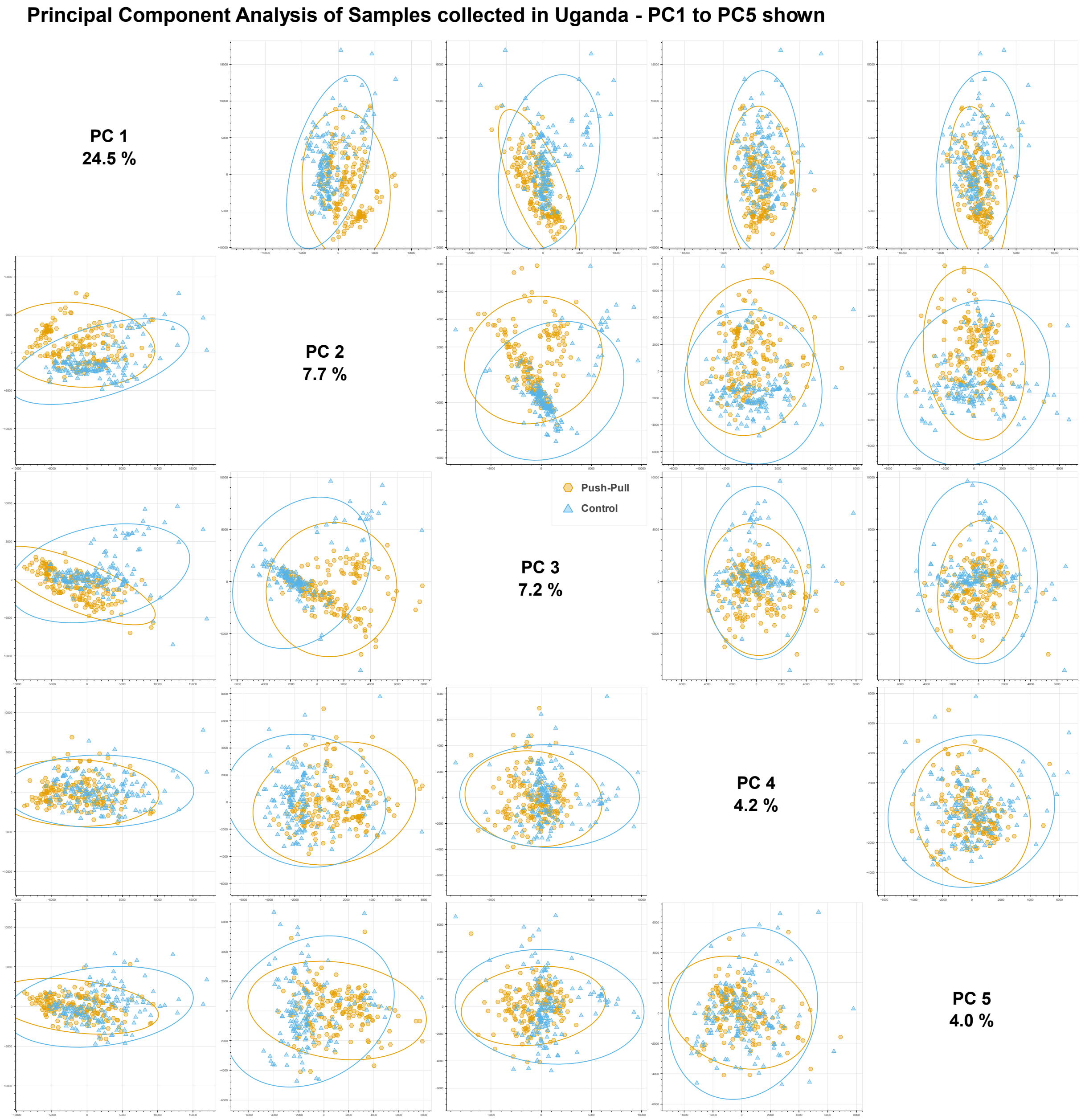

Fig. S4: *Principal component analysis of samples collected in Uganda. In the first five PC dimensions, only a minimal separation of samples from push-pull and control fields can be seen, best visualised in the PC2 against PC3 plot. This is however not a clear enough separation to be confidently used as a basis for compound identification.*

2. Field sampling locations

Supplementary Table S1: *Table of sample collection sites, including field ID, type, code and coordinates, collection and measurement dates and sample count.*

Field_ID	Country	Field_Type	Field_Code	Latitude	Longitude	Collection_Date	Measurement_Date	Sample_Count	Sample_Types	Comment
2101	Kenya	PushPull	KPP_2101	-0.628248409	34.49025823	2022-10-28	2022-11-25	45	Maize, Desmodium, Blank	
2102	Kenya	PushPull	KPP_2102	-0.088838814	34.38689095	2022-11-17	2022-12-08	23	Maize, Blank	
2103	Kenya	PushPull	KPP_2103	-0.050664386	34.63436222	2022-11-07	2022-12-24	45	Maize, Desmodium, Blank	
2104	Kenya	PushPull	KPP_2104	-0.037538478	34.70225331	2022-11-09	2022-12-16	23	Maize, Blank	
2105	Kenya	PushPull	KPP_2105	-0.073618973	34.68236784	2022-11-07	2022-12-16	23	Maize, Blank	
2106	Kenya	PushPull	KPP_2106	-0.031237257	34.65482851	2022-11-10	2022-12-24	45	Maize, Desmodium, Blank	
2107	Kenya	PushPull	KPP_2107	-0.032420097	34.61055031	2022-11-09	2022-12-16	45	Maize, Desmodium, Blank	Desmodium Samples measured 2022-12-22
2108	Kenya	PushPull	KPP_2108	-1.093069263	34.55618169	2022-11-03	2022-12-08	23	Maize, Blank	
2109	Kenya	PushPull	KPP_2109	-1.033645447	34.45003047	2022-11-14	2022-12-27	44	Maize, Desmodium, Blank	
2116	Kenya	PushPull	KPP_2116	-0.573707024	34.32019529	2022-11-16	2022-12-24	23	Maize, Blank	
2117	Kenya	PushPull	KPP_2117	-0.018530126	34.60054232	2022-11-08	2022-12-27	45	Maize, Desmodium, Blank	
2119	Kenya	PushPull	KPP_2119	-1.025940347	34.40922324	2022-11-14	2022-12-30	44	Maize, Desmodium, Blank	
2120	Kenya	PushPull	KPP_2120	-1.021799796	34.48142356	2022-11-02	2022-12-16	24	Maize, Blank	
2126	Kenya	PushPull	KPP_2126	-0.095700641	34.38457232	2022-11-17	2022-12-27	23	Maize, Blank	
2129	Kenya	PushPull	KPP_2129	0.027201749	34.31322421	2022-11-17	2022-12-30	23	Maize, Blank	
2130	Kenya	PushPull	KPP_2130	-0.000187179	34.27050508	2022-11-17	2022-12-08	23	Maize, Blank	
2131	Kenya	PushPull	KPP_2131	-0.047469042	34.35215567	2022-11-17	2022-12-30	23	Maize, Blank	
2133	Kenya	PushPull	KPP_2133	-0.931005712	34.49638714	2022-11-14	2022-12-22	44	Maize, Desmodium, Blank	

Field_ID	Country	Field_Type	Field_Code	Latitude	Longitude	Collection_Date	Measurement_Date	Sample_Count	Sample_Types	Comment
2136	Kenya	PushPull	KPP_2136	-0.642490143	34.51661555	2022-10-28	2022-11-25	45	Maize, Desmodium, Blank	
2140	Kenya	PushPull	KPP_2140	-0.998541924	34.56148921	2022-11-14	2022-12-08	45	Maize, Desmodium, Blank	Desmodium Samples measured 2022-12-22
2201	Kenya	Control	KMC_2201	-0.628620224	34.49033797	2022-10-28	2022-11-25	24	Maize, Blank	
2202	Kenya	Control	KMC_2202	-0.089025455	34.38625183	2022-11-17	2022-12-08	23	Maize, Blank	
2203	Kenya	Control	KMC_2203	-0.050935967	34.63354368	2022-11-07	2022-12-24	24	Maize, Blank	
2204	Kenya	Control	KMC_2204	-0.037734101	34.70243212	2022-11-09	2022-12-16	23	Maize, Blank	
2205	Kenya	Control	KMC_2205	-0.073588451	34.6825185	2022-11-07	2022-12-16	23	Maize, Blank	
2206	Kenya	Control	KMC_2206	-0.032519657	34.65566734	2022-11-10	2022-12-24	24	Maize, Blank	
2207	Kenya	Control	KMC_2207	-0.032712052	34.61021462	2022-11-09	2022-12-16	24	Maize, Blank	
2208	Kenya	Control	KMC_2208	-1.092675977	34.55596272	2022-11-03	2022-12-08	23	Maize, Blank	
2209	Kenya	Control	KMC_2209	-1.033365341	34.44976874	2022-11-14	2022-12-27	23	Maize, Blank	
2216	Kenya	Control	KMC_2216	-0.573143215	34.32004922	2022-11-16	2022-12-24	23	Maize, Blank	
2217	Kenya	Control	KMC_2217	-0.032581003	34.61045916	2022-11-08	2022-12-27	24	Maize, Blank	
2219	Kenya	Control	KMC_2219	-0.070105131	34.32677535	2022-11-14	2022-12-30	23	Maize, Blank	
2220	Kenya	Control	KMC_2220	-1.022193737	34.48161954	2022-11-02	2022-12-16	24	Maize, Blank	
2226	Kenya	Control	KMC_2226	-0.095703969	34.38427466	2022-11-17	2022-12-27	23	Maize, Blank	
2229	Kenya	Control	KMC_2229	0.027021218	34.31344392	2022-11-17	2022-12-30	23	Maize, Blank	
2230	Kenya	Control	KMC_2230	-0.01851015	34.60018072	2022-11-17	2022-12-08	23	Maize, Blank	
2231	Kenya	Control	KMC_2231	-0.047247326	34.35226734	2022-11-17	2022-12-30	23	Maize, Blank	
2233	Kenya	Control	KMC_2233	-0.930915918	34.49626479	2022-11-14	2022-12-22	23	Maize, Blank	
2236	Kenya	Control	KMC_2236	-0.642391598	34.5165088	2022-10-28	2022-11-25	24	Maize, Blank	
2240	Kenya	Control	KMC_2240	-0.570090936	34.37374394	2022-11-14	2022-12-08	24	Maize, Blank	
3106	Rwanda	PushPull	RPP_3106	-1.531497771	30.22710801	2022-12-14	2023-01-19	22	Maize, Blank	
3109	Rwanda	PushPull	RPP_3109	-1.510809565	30.21167358	2022-12-14	2023-01-19	23	Maize, Blank	
3114	Rwanda	PushPull	RPP_3114	-1.510297457	30.21074175	2022-12-19	2023-02-02	23	Maize, Blank	

Field_ID	Country	Field_Type	Field_Code	Latitude	Longitude	Collection_Date	Measurement_Date	Sample_Count	Sample_Types	Comment
3115	Rwanda	PushPull	RPP_3115	-1.530579666	30.2305476	2022-12-14	2023-02-02	18	Maize, Blank	
3117	Rwanda	PushPull	RPP_3117	-1.515026089	30.21325056	2022-12-19	2023-02-02	23	Maize, Blank	
3119	Rwanda	Control	RMC_3119	-1.522441519	30.21838959	2022-12-13	2023-02-10	23	Maize, Blank	
3121	Rwanda	PushPull	RPP_3121	-1.514182034	30.21966501	2022-12-13	2023-02-10	20	Maize, Blank	
3123	Rwanda	Control	RMC_3123	-1.513970346	30.21907446	2022-12-13	2023-02-02	23	Maize, Blank	
3126	Rwanda	PushPull	RPP_3126	-1.514197781	30.21207199	2022-12-14	2023-01-19	23	Maize, Blank	
3132	Rwanda	PushPull	RPP_3132	-1.535121016	30.22895295	2022-12-19	2023-02-02	20	Maize, Blank	
3137	Rwanda	PushPull	RPP_3137	-1.563072206	30.2371913	2022-12-20	2023-01-19	23	Maize, Blank	
3140	Rwanda	PushPull	RPP_3140	-1.542871869	30.22475495	2022-12-19	2023-02-10	23	Maize, Blank	
3204	Rwanda	Control	RMC_3204	-1.5497555	30.22909015	2022-12-20	2023-02-02	20	Maize, Blank	
3205	Rwanda	Control	RMC_3205	-1.550902532	30.22129393	2022-12-20	2023-02-02	22	Maize, Blank	
3206	Rwanda	Control	RMC_3206	-1.531147367	30.22697311	2022-12-14	2023-01-19	23	Maize, Blank	
3211	Rwanda	Control	RMC_3211	-1.562115287	30.23982072	2022-12-20	2023-02-02	23	Maize, Blank	
3221	Rwanda	Control	RMC_3221	-1.513870453	30.21975087	2022-12-13	2023-02-10	22	Maize, Blank	
3226	Rwanda	Control	RMC_3226	-1.513976672	30.21191764	2022-12-14	2023-01-19	23	Maize, Blank	
3337	Rwanda	Control	RMC_3337	-1.563234777	30.23722779	2022-12-20	2023-01-19	22	Maize, Blank	
3509	Rwanda	Control	RMC_3509	-1.511182461	30.21139121	2022-12-14	2023-01-19	23	Maize, Blank	
5101	Uganda	PushPull	UPP_5101	0.73722322	33.12896967	2022-11-26	2023-07-20	23	Maize, Blank	
5106	Uganda	PushPull	UPP_5106	0.724934404	33.09793085	2022-11-26	2023-07-28	23	Maize, Blank	
5111	Uganda	PushPull	UPP_5111	0.729211156	33.16722822	2022-11-20	2023-07-20	23	Maize, Blank	
5112	Uganda	PushPull	UPP_5112	0.725012632	33.17362474	2022-11-21	2023-07-20	23	Maize, Blank	
5123	Uganda	PushPull	UPP_5123	0.828324603	33.24722794	2022-11-25	2023-07-20	23	Maize, Blank	
5126	Uganda	PushPull	UPP_5126	0.815522239	33.27407791	2022-11-25	2023-07-28	23	Maize, Blank	
5141	Uganda	PushPull	UPP_5141	0.967012086	32.99916915	2022-11-23	2023-07-28	22	Maize, Blank	
5161	Uganda	Control	UMC_5161	0.737895764	33.12906087	2022-11-26	2023-07-20	23	Maize, Blank	
5163	Uganda	Control	UMC_5163	0.724961239	33.0977648	2022-11-26	2023-07-28	23	Maize, Blank	During measurements those samples were mislabelled as 5164

Field_ID	Country	Field_Type	Field_Code	Latitude	Longitude	Collection_Date	Measurement_Date	Sample_Count	Sample_Types	Comment
5168	Uganda	Control	UMC_5168	0.729265642	33.16743768	2022-11-21	2023-07-20	23	Maize, Blank	
5169	Uganda	Control	UMC_5169	0.724763967	33.17316017	2022-11-20	2023-07-20	23	Maize, Blank	
5173	Uganda	Control	UMC_5173	0.828148253	33.24707203	2022-11-25	2023-07-20	23	Maize, Blank	
5174	Uganda	Control	UMC_5174	0.810330857	33.27250888	2022-11-25	2023-07-28	23	Maize, Blank	
5188	Uganda	Control	UMC_5188	0.967143249	32.99990039	2022-11-24	2023-07-28	23	Maize, Blank	
icipe	Kenya	PushPull	KPP_icipe	-0.430020071	34.20786815	2022-11-18	2022-12-30	15	BorderGrasses	

3. Target Compound analysis

3.1. MS/MS Fragment Analysis

Supplementary Table S2: *Overview of target features and the annotations based on the Sirius / Canopus workflow.*

ID	RT [min]	*m/z* [Da]	Ion Adduct	Neutral Mass [Da]	Sum formula	Main Class	Secondary Class	First Proposed Structure	Second Proposed Structure
**#1**	1.79	358.11326	$[M+H]^+$	357.10598	$C_{15}H_{19}NO_9$	Hexose glycosides	Benzoxa-zinones	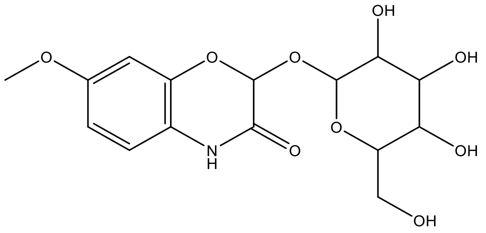	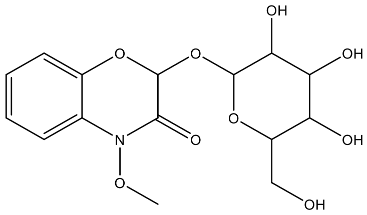
**#2**	2.24	166.04991	$[M+H]^+$	165.04259	$C_8H_7NO_3$	Benzoxa-zolones	Anisoles	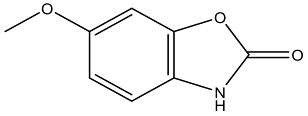	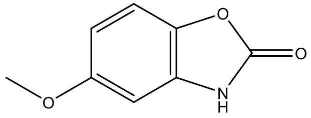

**#3**	2.24	194.04479	$[M+H]^+$	193.03751	$C_9H_7NO_4$	Benzoxa-zolones	Anisoles	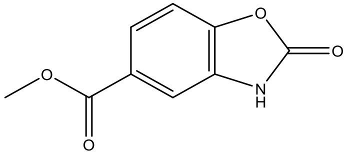	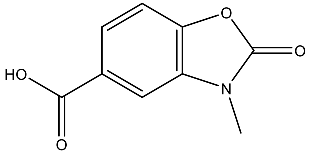
**#4**	2.24	226.07098	$[M+H]^+$	225.06372	$C_{10}H_{11}NO_5$	Benzoxa-zines	Anisoles	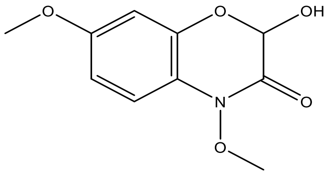	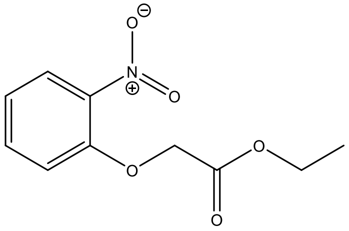
**#5**	2.23	388.12366	$[M+H]^+$	387.11655	$C_{16}H_{21}NO_{10}$	Benzoxa-zinones	O-glycosyl compounds	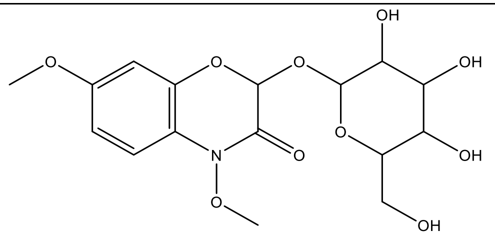	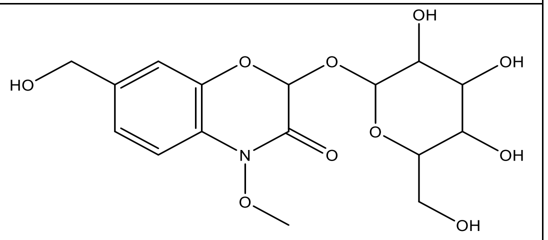
**#6**	2.23	405.15033	$[M+NH_4]^+$	387.1165	$C_{16}H_{21}NO_{10}$	Hexose glycosides	Benzoxa-zinones	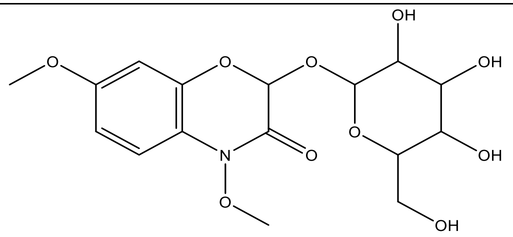	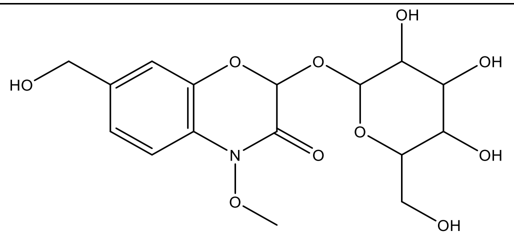
**#7**	2.24	432.11453	$[M+COOH]^-$	387.11633	$C_{16}H_{21}NO_{10}$	Hexose glycosides	Benzoxa-zinones	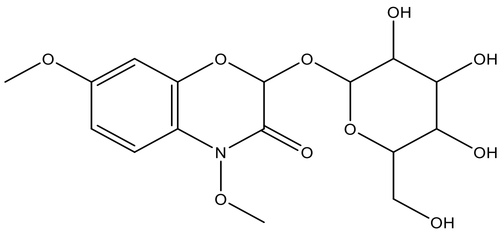	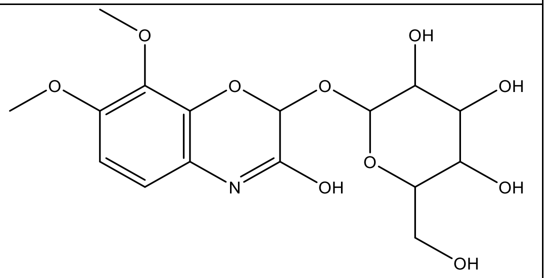

3.2. Abundances of target ions

Supplementary Table S3: *Results of a t-test of the target features in each single country dataset including fold change and max. intensity values. For the filtering procedure (p < 0.005) the uncorrected p-values were used, and after a false discovery rate (FDR) correction all values remain below 0.01 with only two ions in the Rwandan dataset rising above the 0.005 cutoff. p-values listed as 0 are rounded values below 10^{-6}.*

**Kenya**	**Ion ID**	**p-Value**	**Fold Change "PP"/"Con"**	**p-Value(FDR)**	**Max. Intensity**
HMBOA-Glc	#1	0.000048	1.521	0.00017	247275
2.24 min : 165.04263 Da	#2	0	2.027	0	1178557
2.24 min : 193.03752 Da	#3	0	1.961	0	555702
HDMBOA	#4	0	2.019	0	1456608
HDMBOA-Glc proton adduct	#5	0	2.044	0	937090
HDMBOA-Glc ammonium add	#6	0.000138	1.822	0.000455	733058
HDMBOA-Glc formate adduct	#7	0	1.978	0	1513777
**Rwanda**	**Ion ID**	**p-Value**	**Fold Change "PP"/"Con"**	**p-Value(FDR)**	**Max. Intensity**
HMBOA-Glc	#1	0.000973	1.65	0.005746	68698
2.24 min : 165.04263 Da	#2	0	1.771	0.000008	352583
2.24 min : 193.03752 Da	#3	0	1.725	0.000008	140775
HDMBOA	#4	0.000001	1.647	0.000027	271454
HDMBOA-Glc proton adduct	#5	0.000006	1.771	0.000099	178139
HDMBOA-Glc ammonium add	#6	0.001438	1.711	0.007796	84974
HDMBOA-Glc formate adduct	#7	0	1.884	0.000003	360363
**Uganda**	**Ion ID**	**p-Value**	**Fold Change "PP"/"Con"**	**p-Value(FDR)**	**Max. Intensity**
HMBOA-Glc	#1	0.00002	3.021	0.000059	126699
2.24 min : 165.04263 Da	#2	0	4.916	0	23518
2.24 min : 193.03752 Da	#3	0	5.611	0	270082
HDMBOA	#4	0	5.429	0	543847
HDMBOA-Glc proton adduct	#5	0	9.352	0.000002	443395
HDMBOA-Glc ammonium add	#6	0.000003	15.016	0.000011	259940
HDMBOA-Glc formate adduct	#7	0	7.226	0	1436806

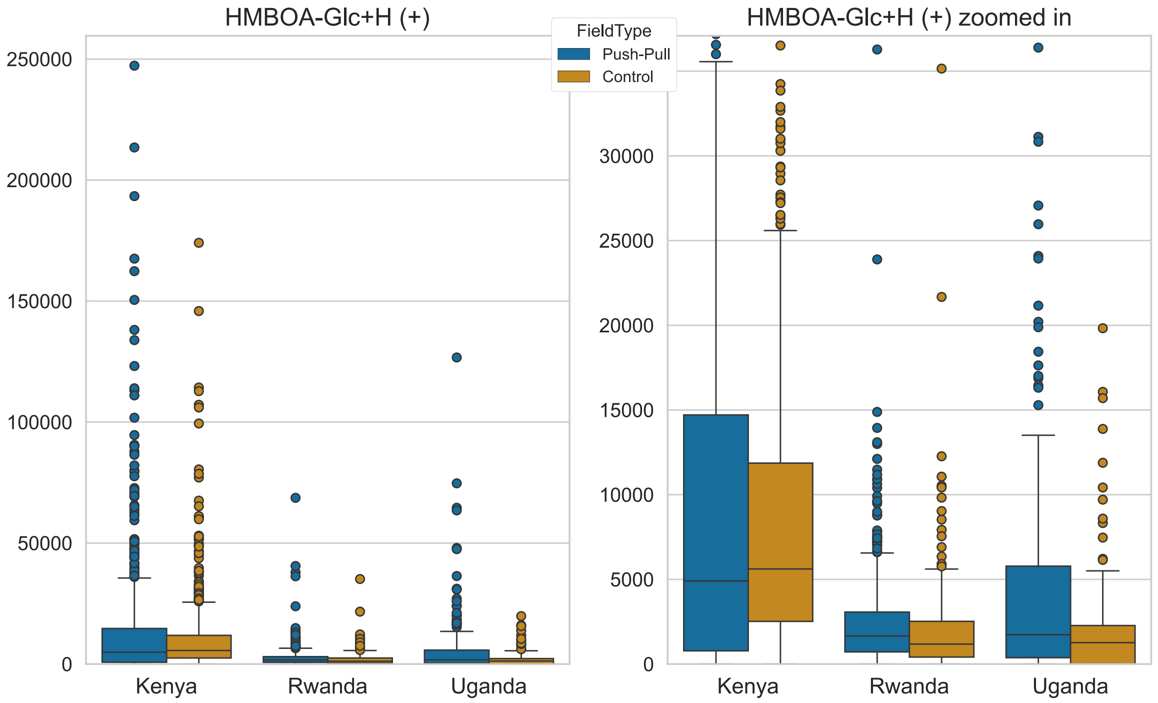

Fig. S5: *Boxplot of the abundance of target feature #1 (HMBOA-Glc, [M+H]$^+$ ion, 358.11326 m/z) by country and field type. The right side shows a zoomed view to better visualise the differences in the samples from Rwanda and Uganda.*

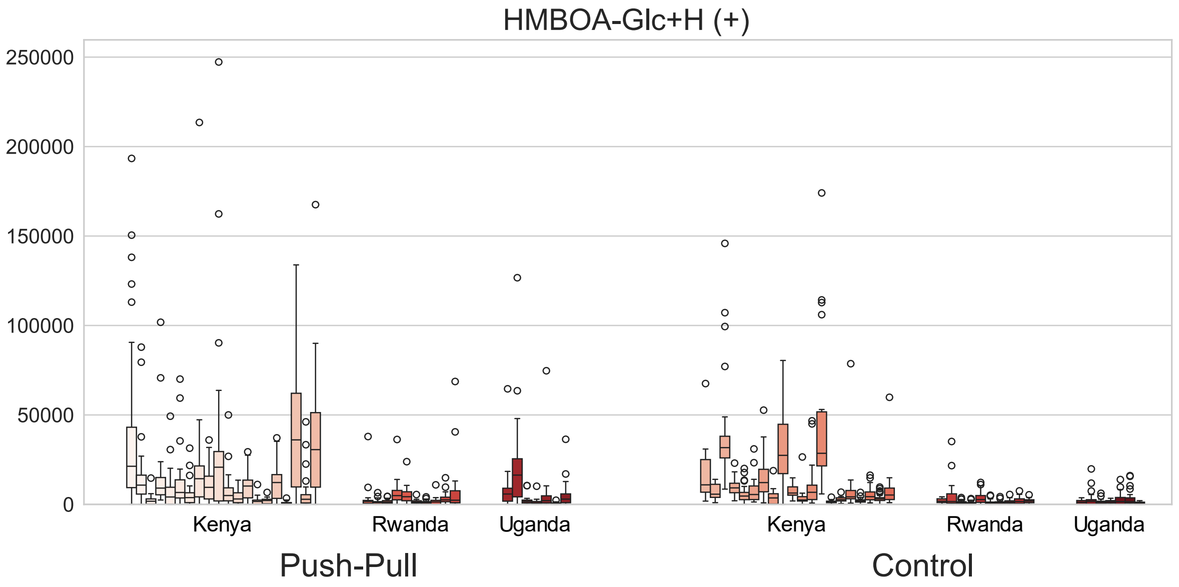

Fig. S6: *Boxplot of the abundance of target feature #1 (HMBOA-Glc, [M+H]$^+$ ion, 358.11326 m/z) by country, field type, and field ID. The large number of outliers observed in* ***Fig. S5*** *can sometimes be linked to individual field sites with extraordinarily high abundances.*

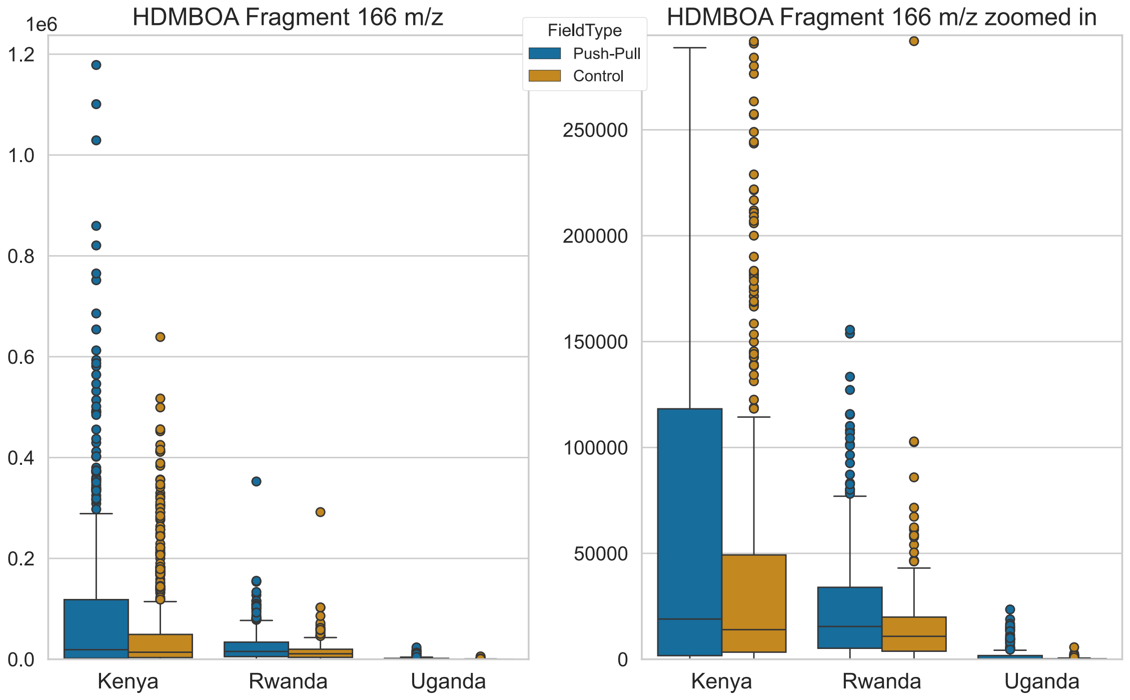

Fig. S7: *Boxplot of the abundance of target feature #2 (HDMBOA fragment, 166.04991 m/z) by country and field type. The right side shows a zoomed view to better visualise the differences in the samples from Rwanda and Uganda.*

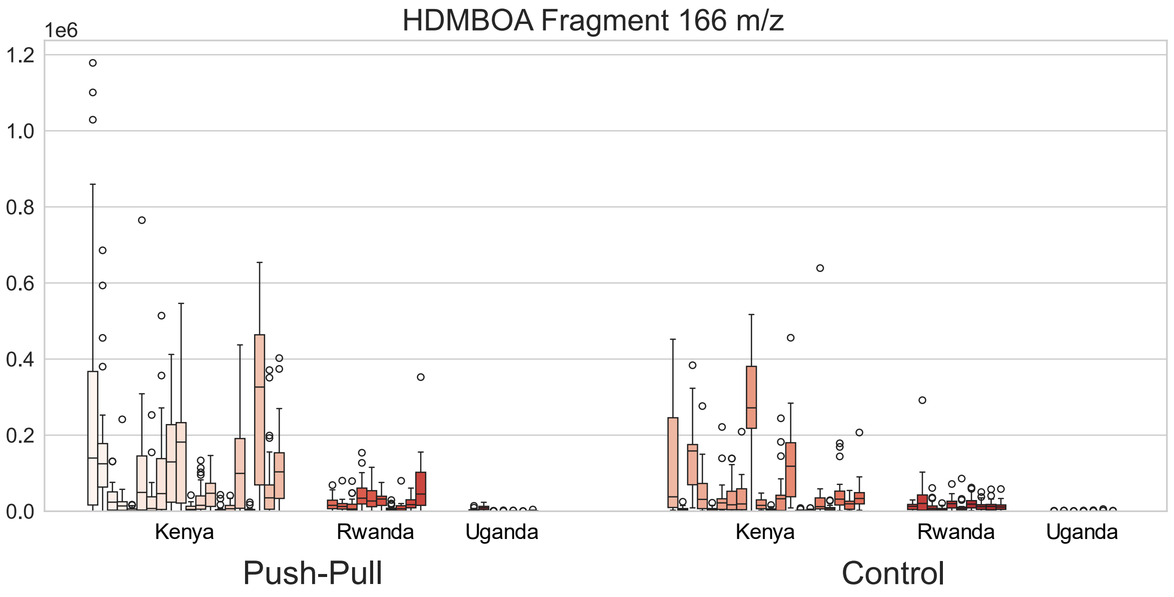

Fig. S8: *Boxplot of the abundance of target feature #2 (HDMBOA fragment, 166.04991 m/z) by country, field type, and field ID. The large number of outliers observed in* ***Fig. S7*** *can sometimes be linked to individual field sites with extraordinarily high abundances.*

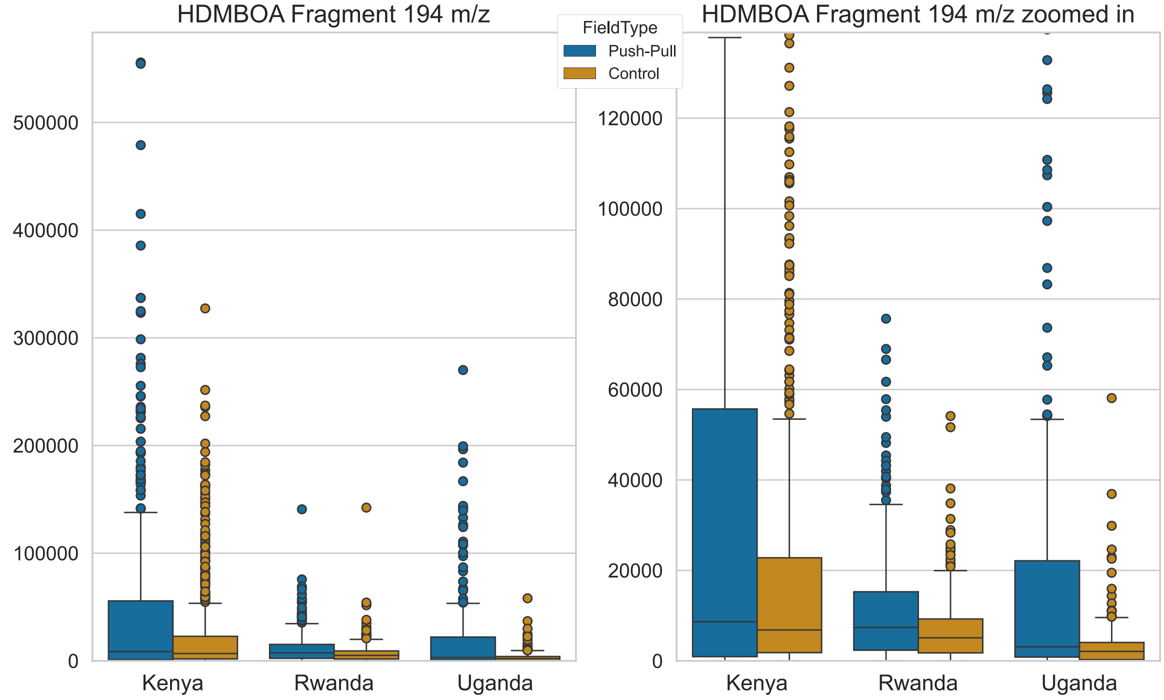

Fig. S9: *Boxplot of the abundance of target feature #3 (HDMBOA fragment, 194.04479 m/z) by country and field type. The right side shows a zoomed view to better visualise the differences in the samples from Rwanda and Uganda.*

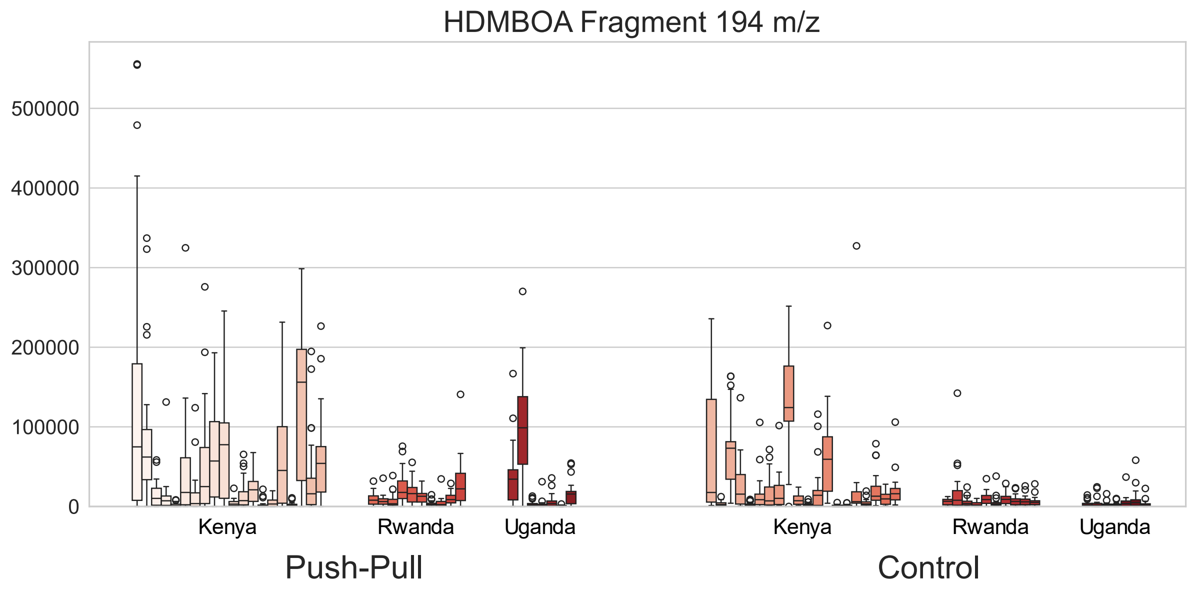

Fig. S10: *Boxplot of the abundance of target feature #3 (HDMBOA fragment, 194.04479 m/z) by country, field type, and field ID. The large number of outliers observed in **Fig. S9** can sometimes be linked to individual field sites with extraordinarily high abundances.*

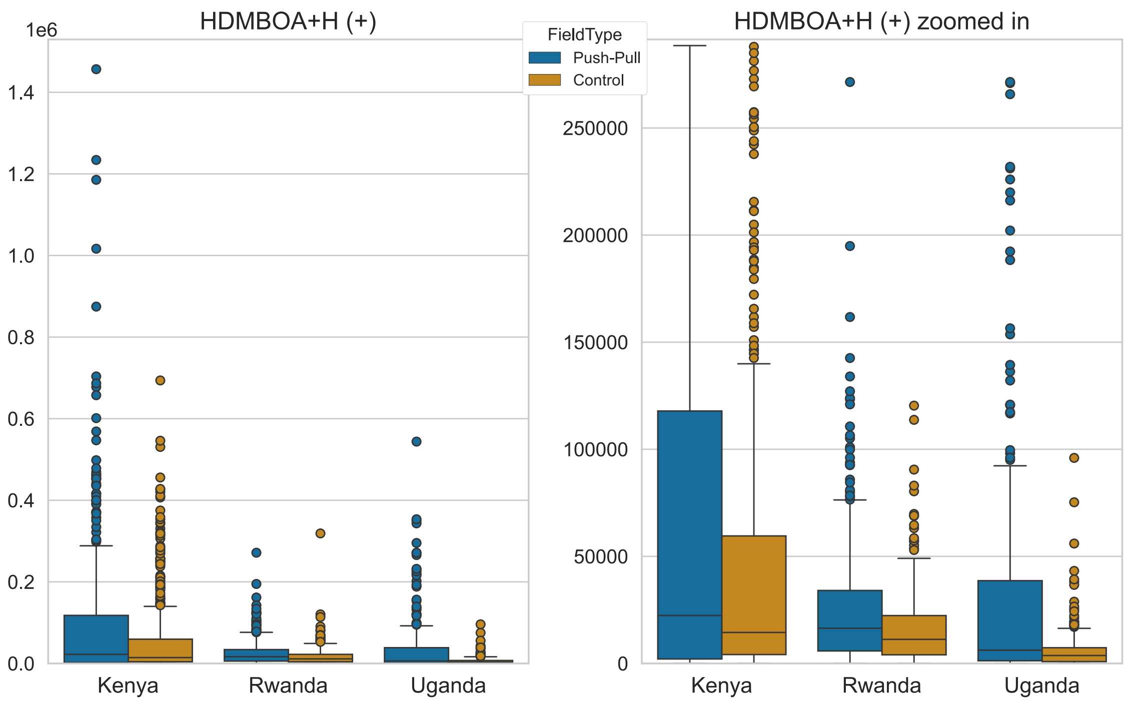

Fig. S11: *Boxplot of the abundance of target feature #4 (HDMBOA, $[M+H]^+$ ion, 226.07098 m/z) by country and field type. The right side shows a zoomed view to better visualise the differences in the samples from Rwanda and Uganda.*

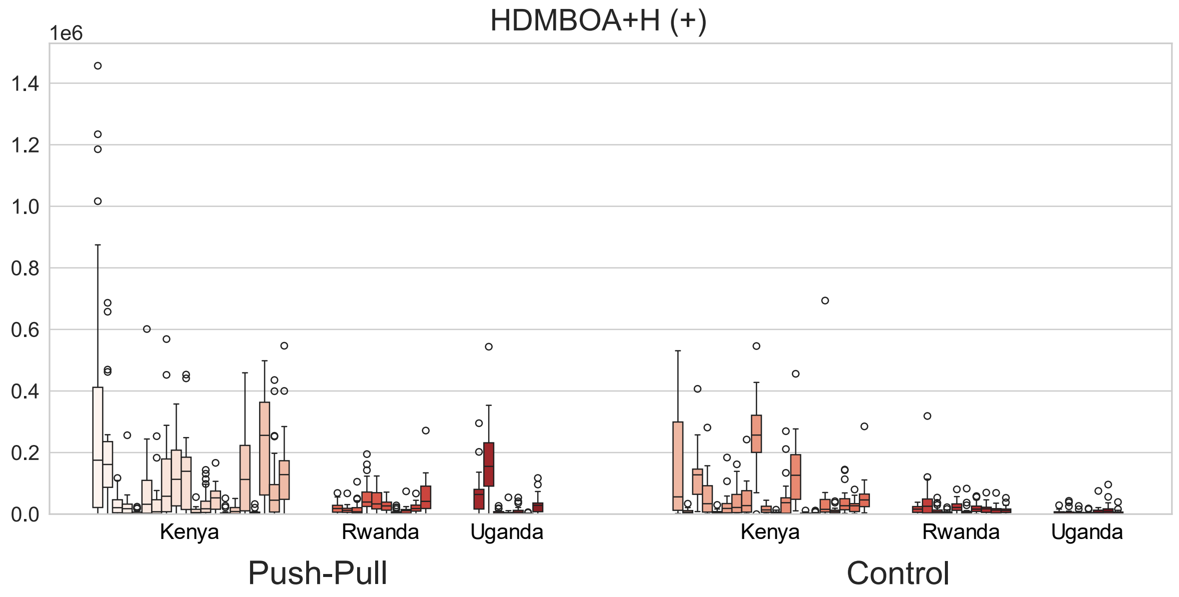

Fig. S12: *Boxplot of the abundance of target feature #4 (HDMBOA, $[M+H]^+$ ion, 226.07098 m/z) by country, field type, and field ID. The large number of outliers observed in* ***Fig. S11*** *can sometimes be linked to individual field sites with extraordinarily high abundances.*

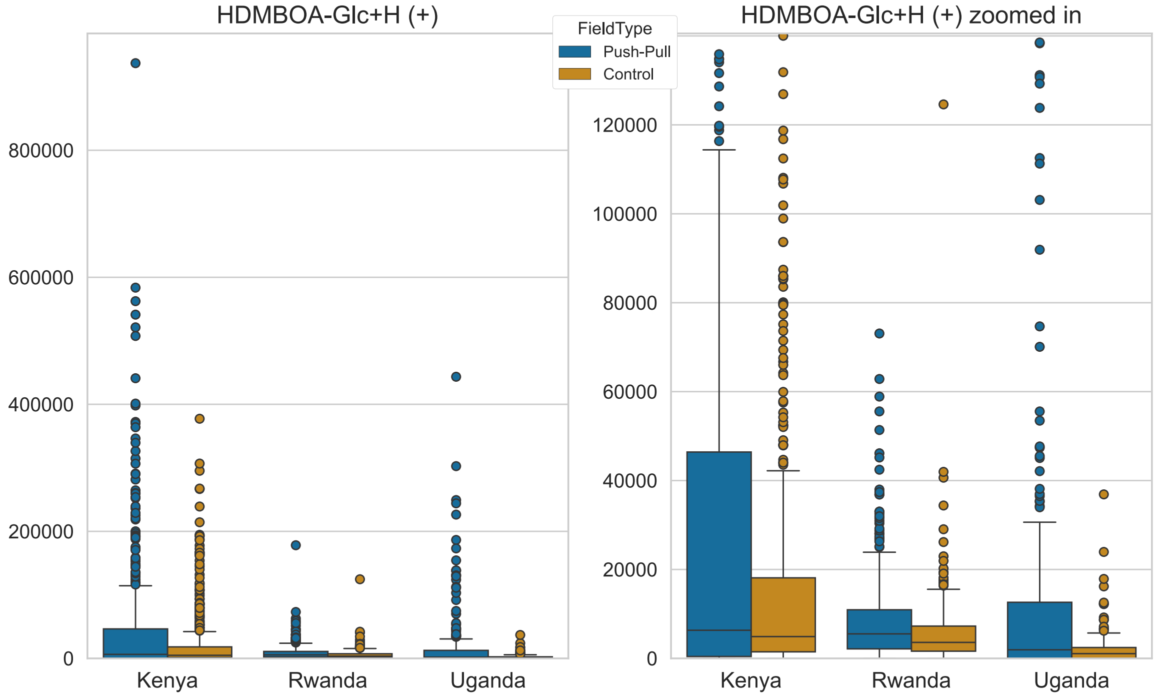

Fig. S13: *Boxplot of the abundance of target feature #5 (HDMBOA-Glc, [M+H]⁺ ion, 388.12366 m/z) by country and field type. The right side shows a zoomed view to better visualise the differences in the samples from Rwanda and Uganda.*

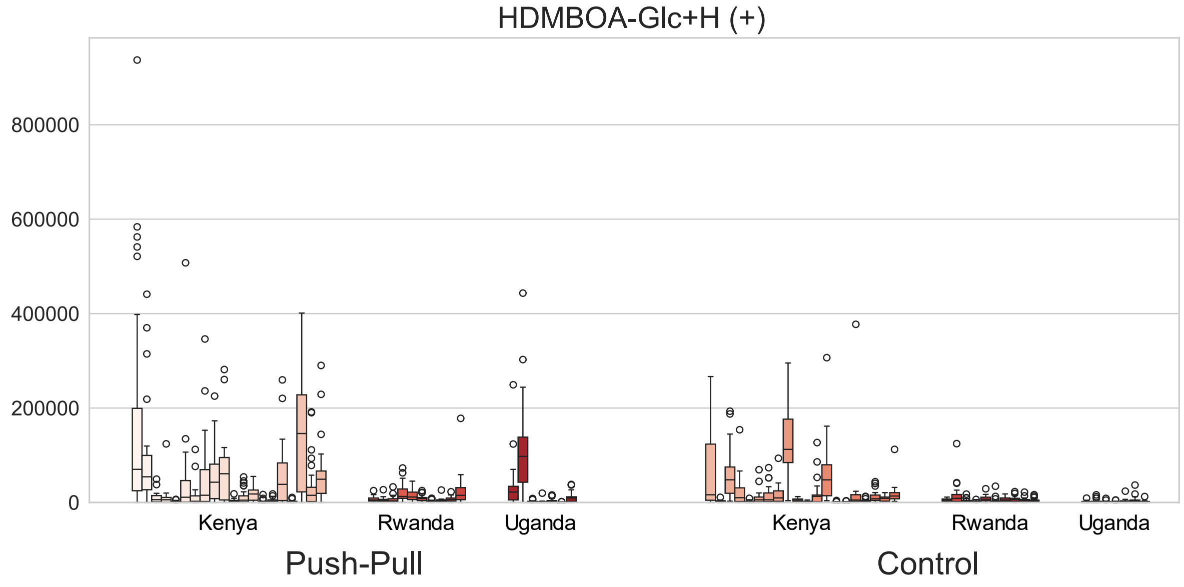

Fig. S14: *Boxplot of the abundance of target feature #5 (HDMBOA-Glc, [M+H]⁺ ion, 388.12366 m/z) by country, field type, and field ID. The large number of outliers observed in **Fig. S13** can sometimes be linked to individual field sites with extraordinarily high abundances.*

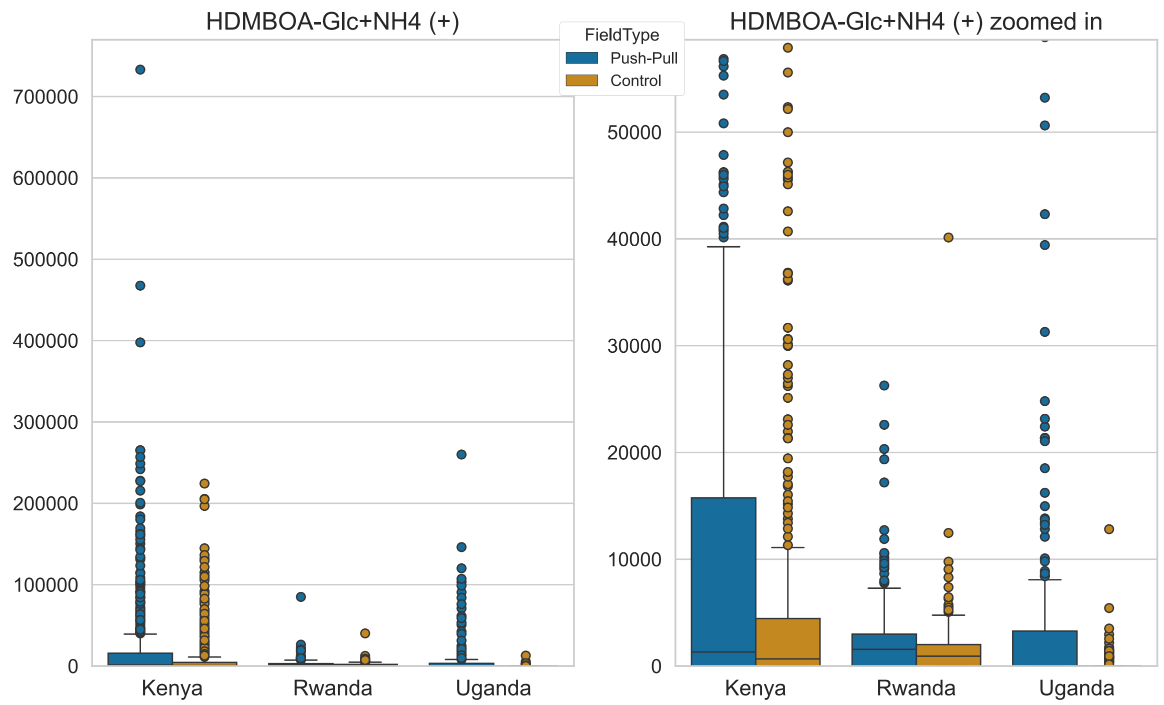

Fig. S15: *Boxplot of the abundance of target feature #6 (HDMBOA-Glc, $[M+NH_4]^+$ ion, 405.15033 m/z) by country and field type. The right side shows a zoomed view to better visualise the differences in the samples from Rwanda and Uganda.*

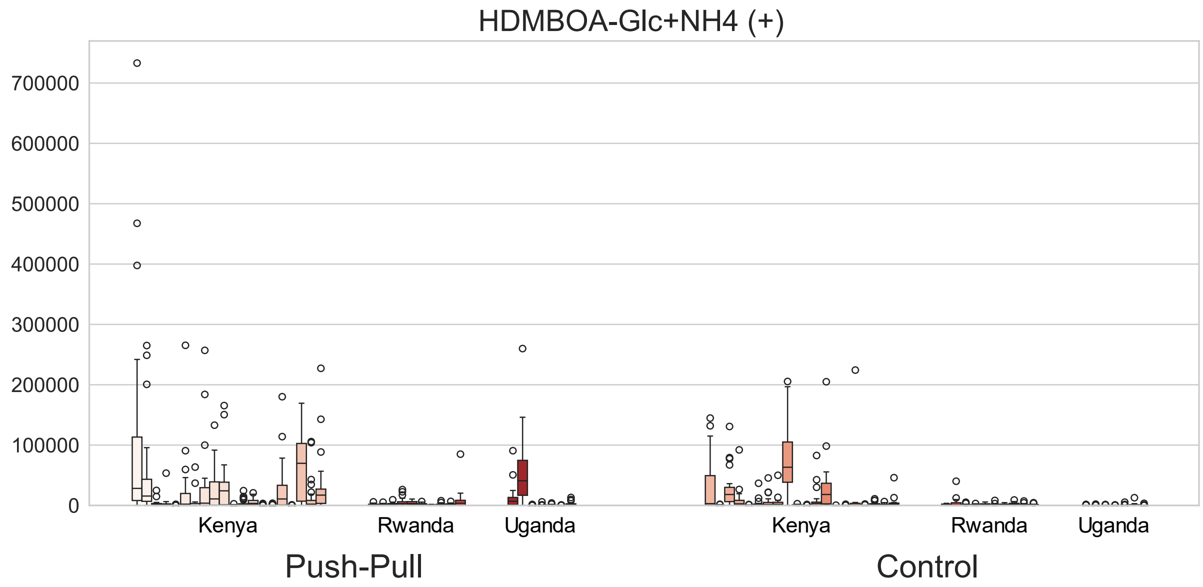

Fig. S16: *Boxplot of the abundance of target feature #6 (HDMBOA-Glc, $[M+NH_4]^+$ ion, 405.15033 m/z) by country, field type, and field ID. The large number of outliers observed in **Fig. S15** can sometimes be linked to individual field sites with extraordinarily high abundances.*

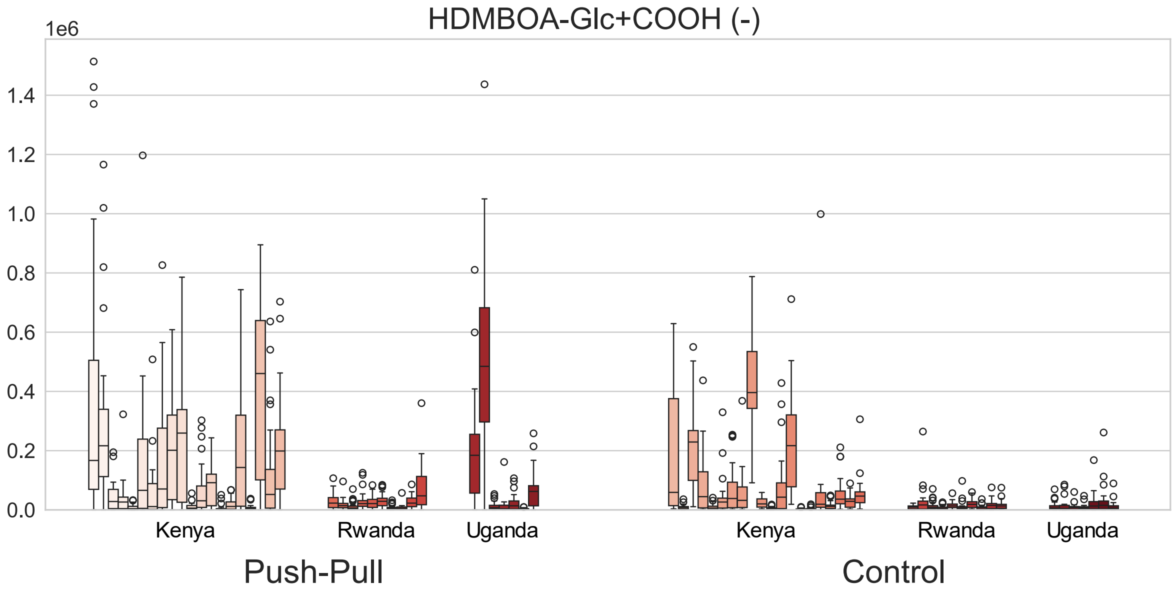

Fig. S17: *Boxplot of the abundance of target feature #7 (HDMBOA-Glc, [M+COOH]⁻ ion, 432.11453 m/z) by country, field type, and field ID. The large number of outliers observed in* ***Fig. 4*** *(main text) can sometimes be linked to individual field sites with extraordinarily high abundances.*

4. Isolation of HDMBOA-Glc – detailed methodology

The first step of the isolation was using a Shimadzu Prominence semi-preparative RP-HPLC system (Shimadzu, Japan) with a CBM-40 system controller module, an FRC-10A fraction collector, two LC-20AR pumps, and an SPD-40 UV/VIS detector, using a Zorbax 300SB-C18 Semi-Preparative column (9.4 × 250 mm, 5 µm particle size, *Agilent*, Santa Clara, CA, USA) kept at 23 °C, with a flow rate of 3.5 mL/min. A binary solvent system was used, with H_2O containing 0.1% TFA as solvent A, and MeCN containing 0.1% TFA as solvent B. The gradient was as follows: (I) initial hold at 3% B for 5 minutes (II) linear increase to 40% B in at minute 42, (III) linear increase to 75% B at minute 54, (IV) linear increase to 95%B at minute 56, (V) wash column with 95% B for 9 minutes.

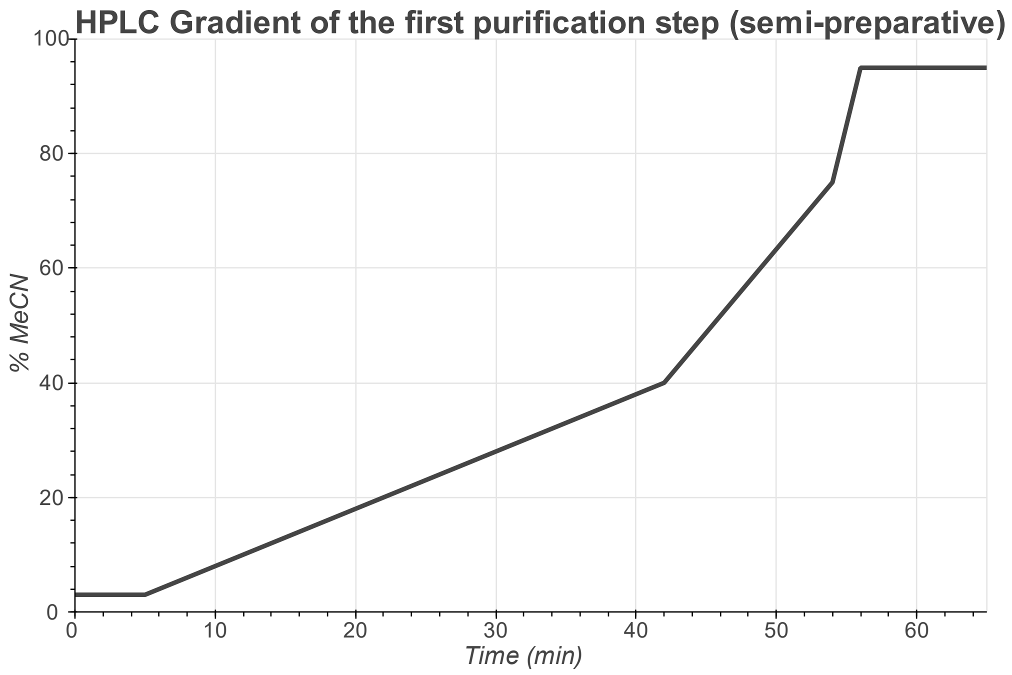

Fig. S18: Solvent gradient for the semi-preparative isolation of HDMBOA-Glc from pooled samples.

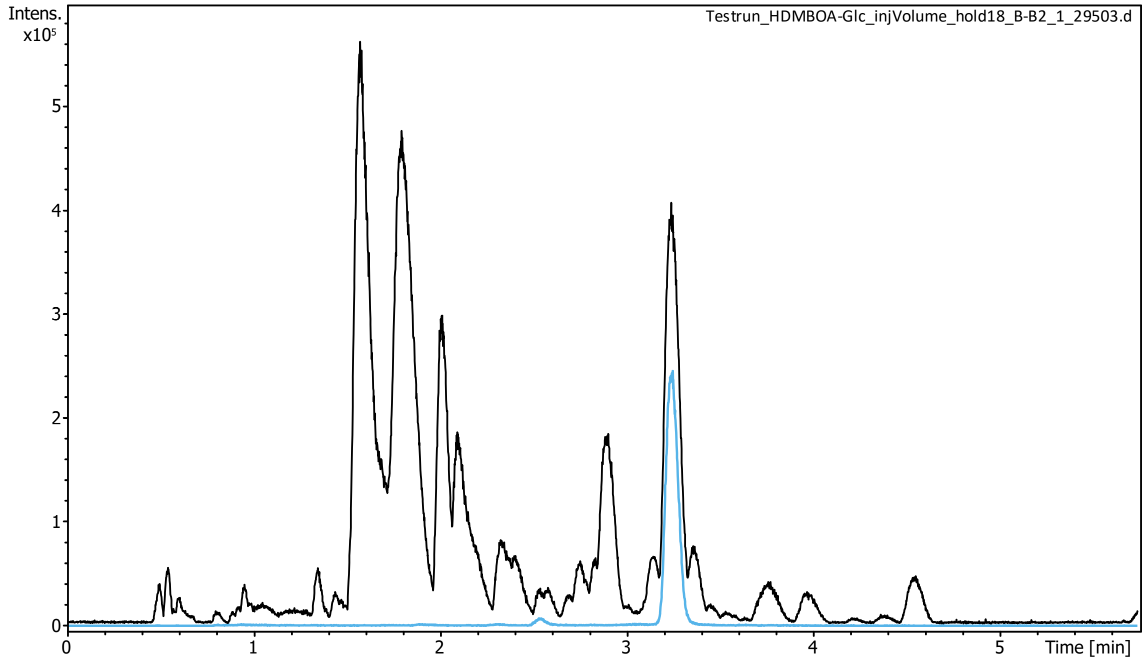

Fig. S19: Chromatogram of the fraction containing HDMBOA-Glc after the semi-preparative isolation, showing the BPC (black) and the EIC trace for HDMBOA-Glc (388.1228 m/z, blue).

The second purification step was performed on an ACQUITY Premier HSS T3 (2.1 × 100 mm, 1.7 µm particle size, *Waters*, Milford, MA, USA) column, using the Vanquish Horizon UHPLC System mentioned used for UHPLC-MS measurements with H_2O containing 0.1% FA as solvent A, and MeCN containing 0.1% FA as solvent B at 30 °C. The isolation was done with a constant flow of 0.45 mL/min, using a gradient as follows: (I) initial hold at 17% B for 1

minute, (II) linear increase to 18% B until minute 2.5, (III) hold at 18% B for 1 minute, (IV) linear increase to 19% B at minute 3.7, (V) linear increase to 100 %B at minute 4.2, (VI) wash at 100% B for 3.8 minutes, followed by reconditioning at 17% B for 2 minutes for a total runtime of 10 minutes.

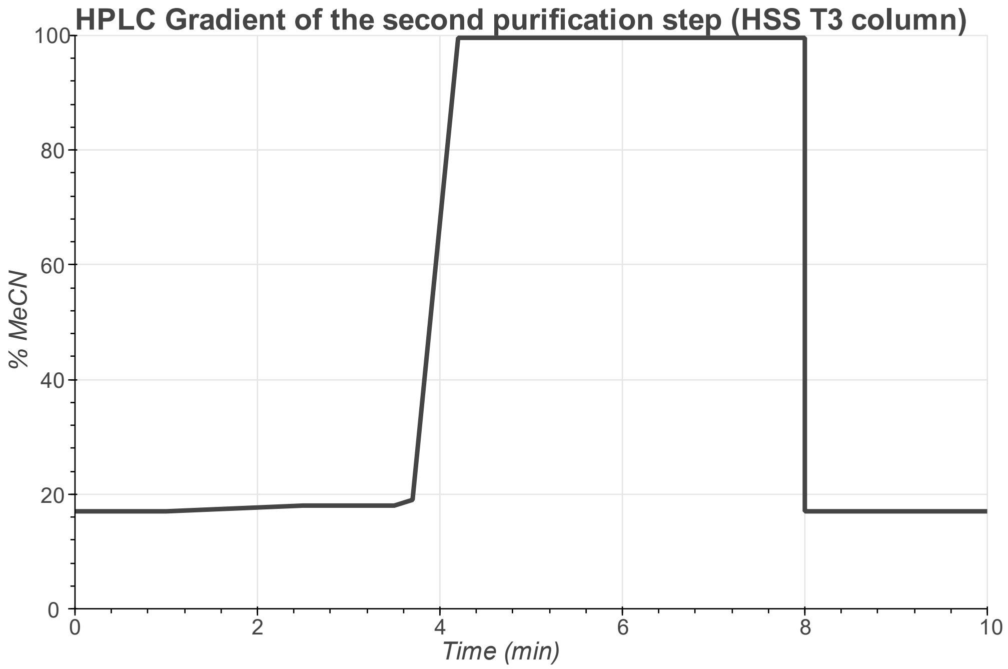

Fig. S20: *Solvent gradient for the second isolation step of HDMBOA-Glc using an analytical HSS T3 column.*

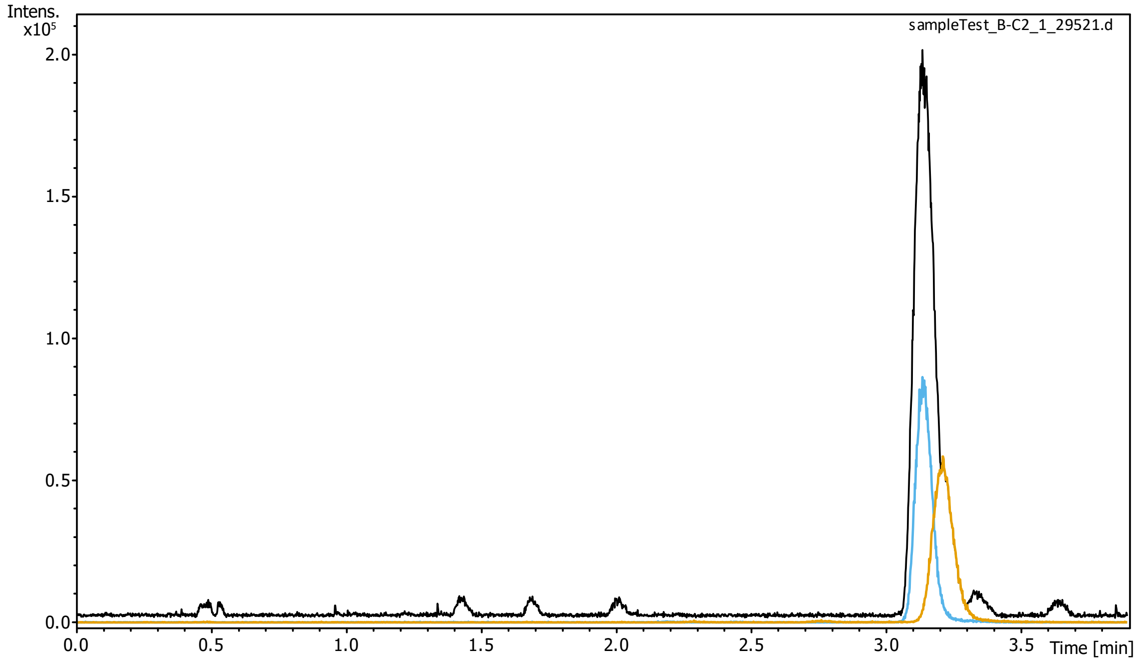

Fig. S21: *Chromatogram of HDMBOA-Glc after the second isolation step, showing the BPC (black) and EIC traces for HDMBOA-Glc (388.1228 m/z, blue) and an unidentified contaminant (579.1697 m/z, yellow).*

The third purification step was done on an Accucore Phenyl-X (2.1 × 100 mm, 2.6 µm particle size, *Thermo Fisher*, Waltham, MA, USA) column using the Vanquish Horizon UHPLC System mentioned used for UHPLC-MS measurements with H_2O containing 0.1% FA as solvent A, and MeCN containing 0.1% FA as solvent B at 30 °C. The isolation was done with a constant flow of 0.5 mL/min, using a gradient as follows: (I) linear increase from 15% B to 20 %B in 3 minutes, (II) jump to 100% B and was for 2 minutes, (III) jump to 15% B and recondition for 2 minutes for a total runtime of 7 minutes.

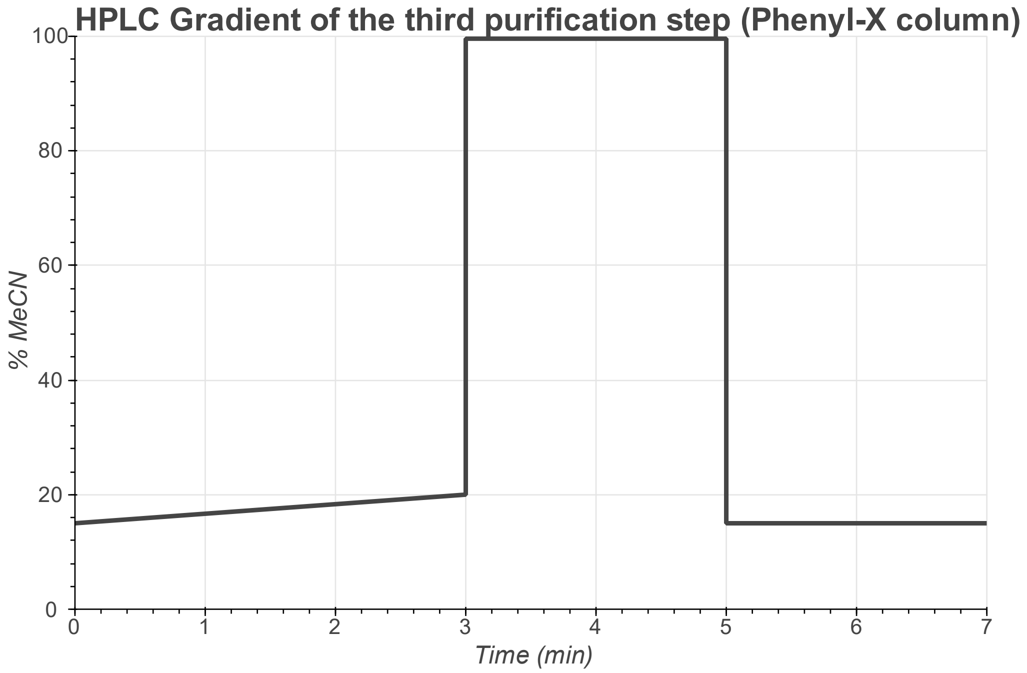

Fig. S22: *Solvent gradient for the third isolation step of HDMBOA-Glc using an analytical Phenyl-X column.*

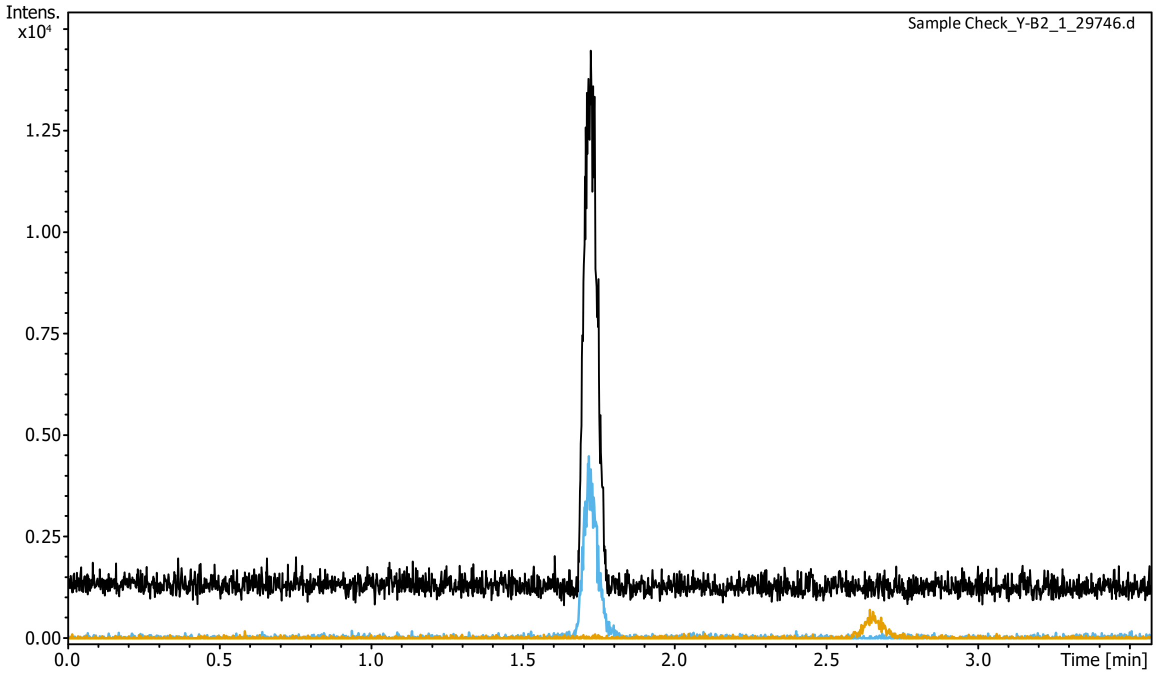

Fig. S23: *Chromatogram of HDMBOA-Glc after the third isolation step, showing the BPC (black) and EIC traces for HDMBOA-Glc (388.1228 m/z, blue) and an unidentified contaminant (579.1697 m/z, yellow).*

5. NMR analysis of HDMBOA-Glc

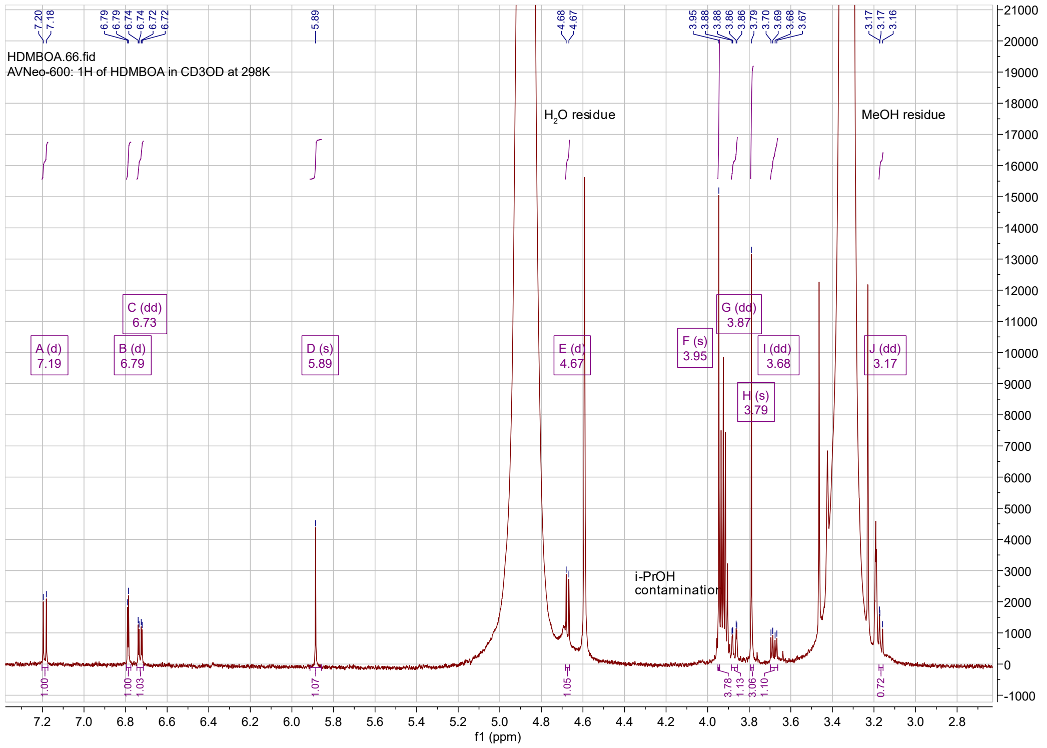

Fig. S24: *Proton NMR spectrum of HDMBOA-Glc with peak integrals and marked solvent residues.*

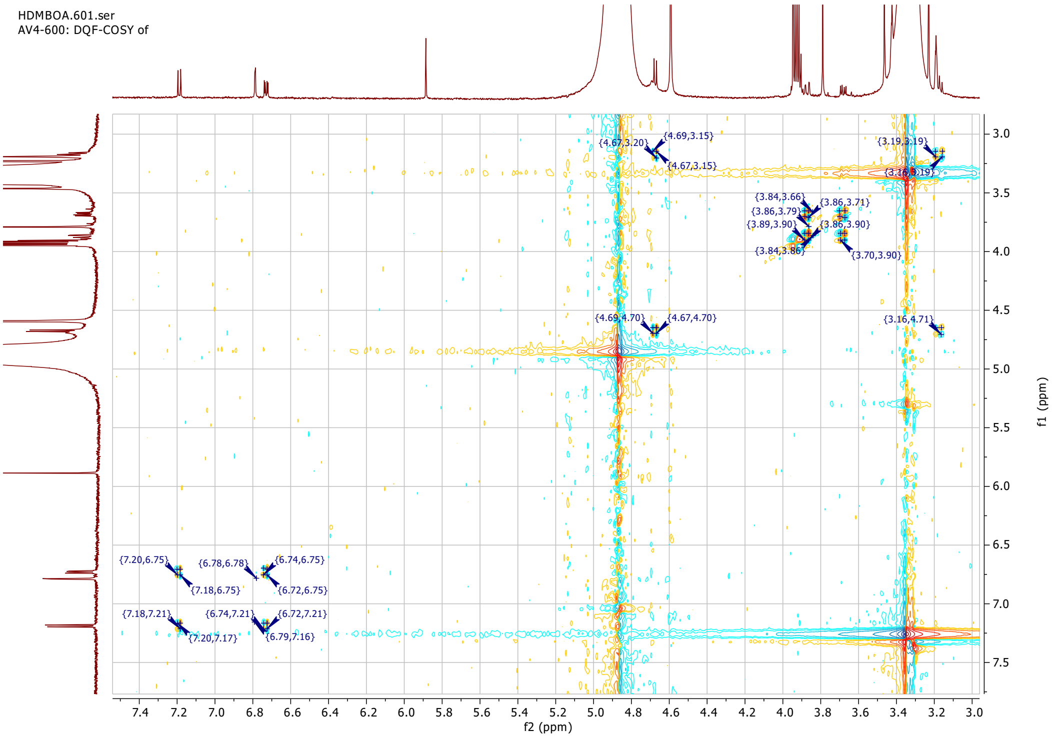

Fig. S25: *Two-dimensional correlation spectroscopy (COSY) spectrum of HDMBOA-Glc with marked peaks.*

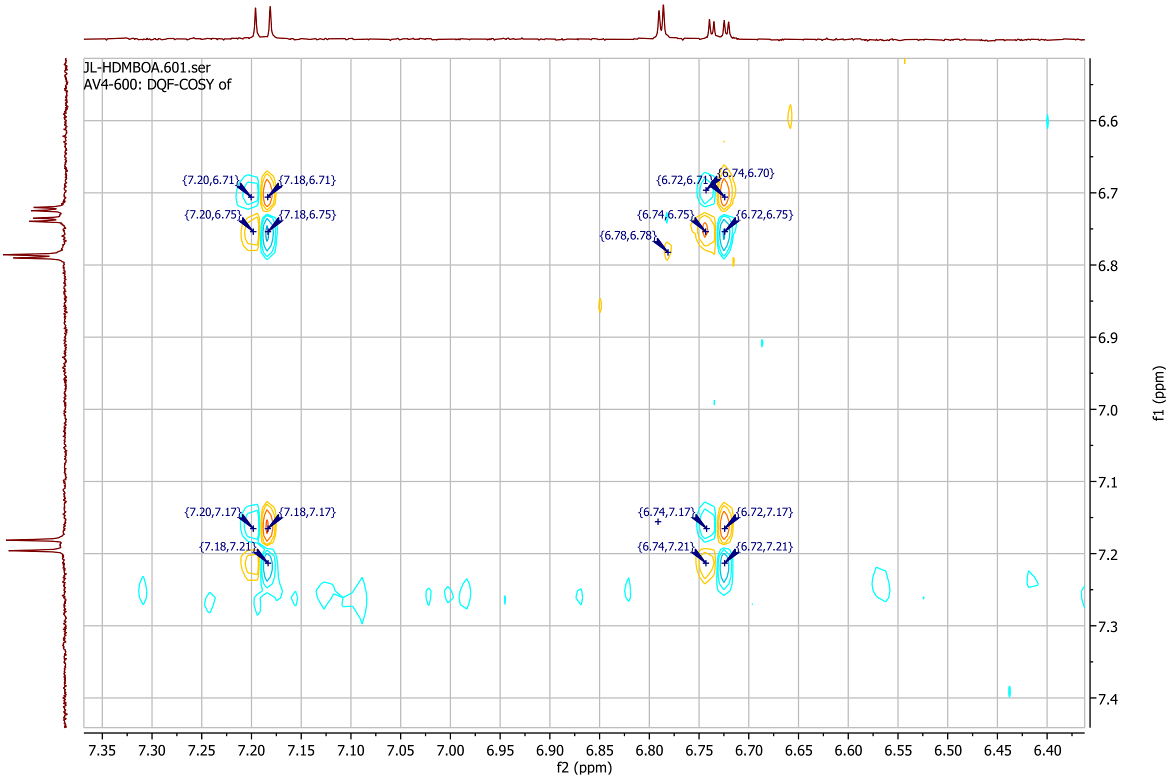

Fig. S26: *Zoomed view of the aromatic region of the COSY spectrum shown in* ***Fig. S25****.*

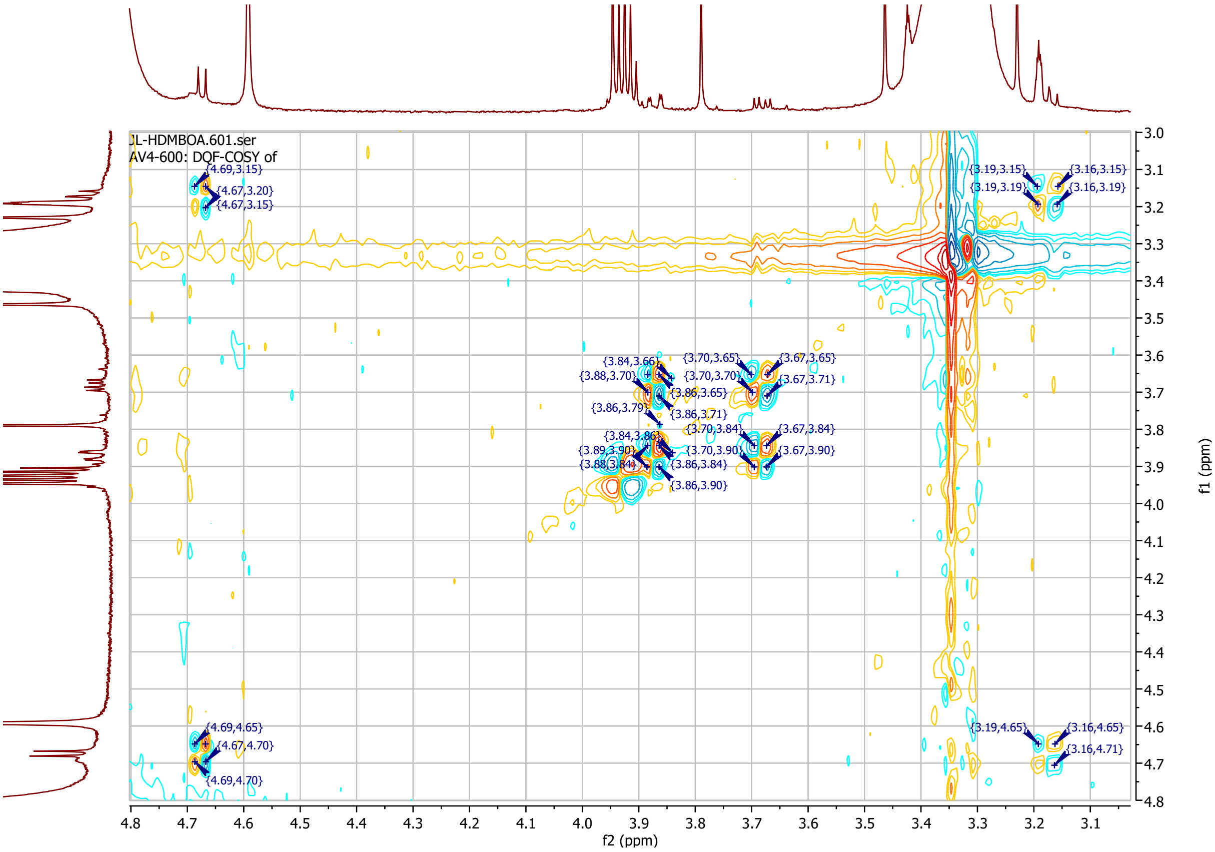

Fig. S27: *Zoomed view of the non-aromatic region of the COSY spectrum shown in* ***Fig. S25****.*

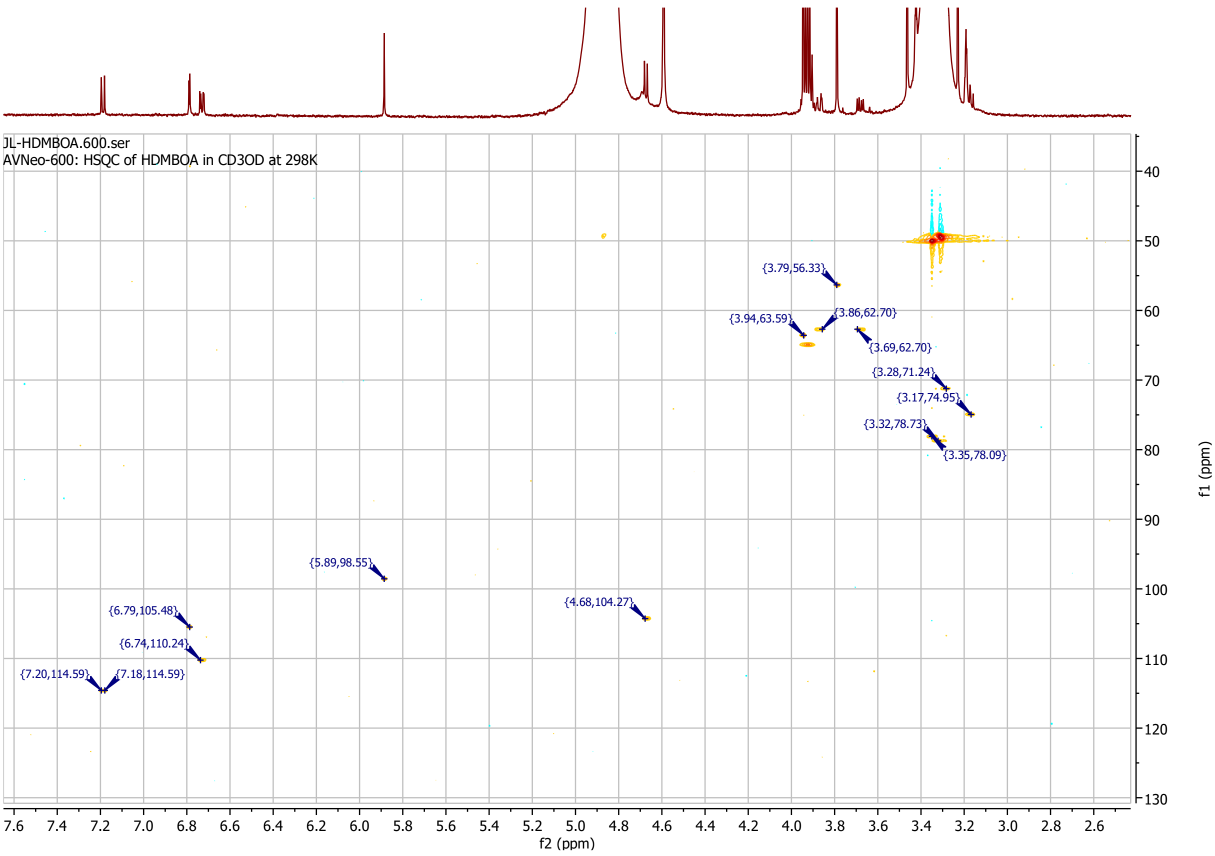

Fig. S28: *Heteronuclear single quantum coherence spectroscopy (HSQC) spectrum of HDMBOA-Glc.*

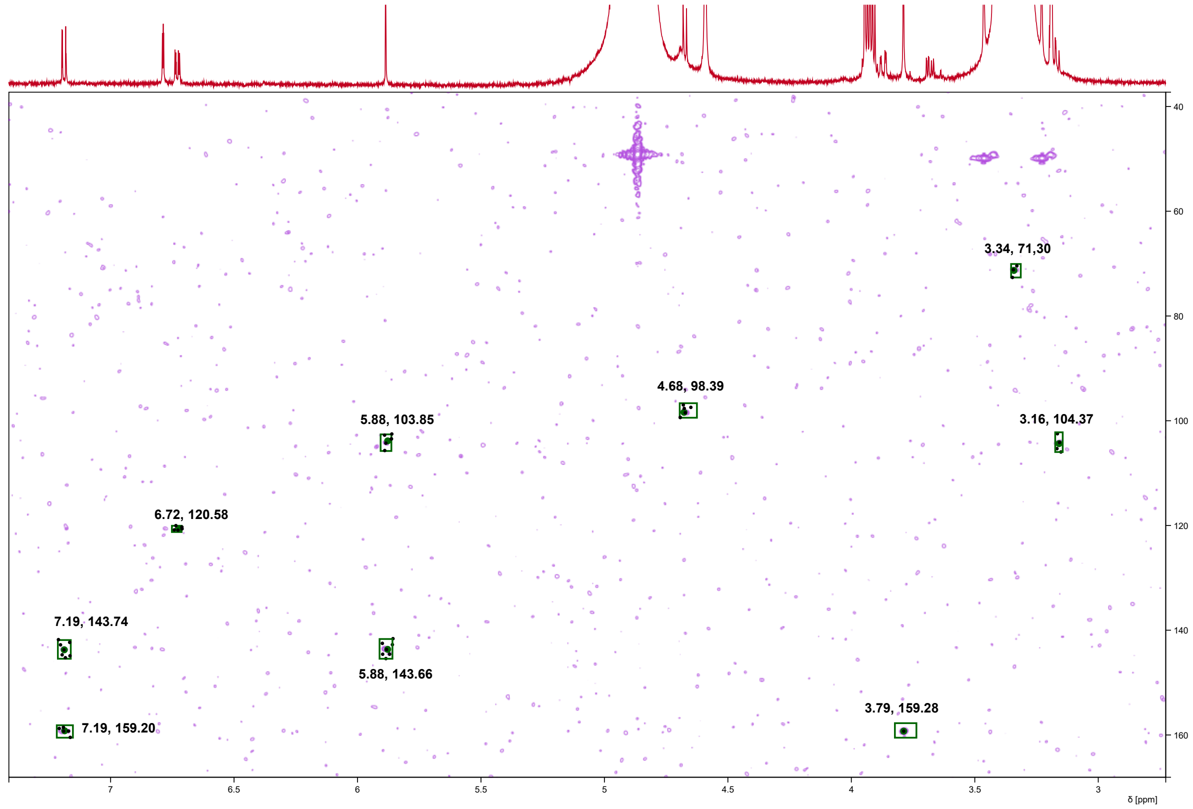

Fig. S29: *Heteronuclear Multiple Bond Correlation (HMBC) spectrum of HDMBOA-Glc. This spectrum was processed using NMRium (https://www.nmrium.org) instead of the previously used MestReNova due to different zoom levels.*
